# Supplementary material for: Treatment with a Gamma-Secretase Inhibitor Promotes Functional Recovery in Human iPSC- Derived Transplants for Chronic Spinal Cord Injury
Source: Stem Cell Reports. 2018 Nov 29;11(6):1416–32. doi: 10.1016/j.stemcr.2018.10.022 (PMC6294244; doi:10.1016/j.stemcr.2018.10.022)
Supplement: Document S1. Supplemental Experimental Procedures and Figures S1–S4 [file mmc1.pdf]

**Stem Cell Reports, Volume *11***

## Supplemental Information

# Treatment with a Gamma-Secretase Inhibitor Promotes Functional Recovery in Human iPSC- Derived Transplants for Chronic Spinal Cord Injury

**Toshiki Okubo, Narihito Nagoshi, Jun Kohyama, Osahiko Tsuji, Munehisa Shinozaki, Shinsuke Shibata, Yoshitaka Kase, Morio Matsumoto, Masaya Nakamura, and Hideyuki Okano**

**A**

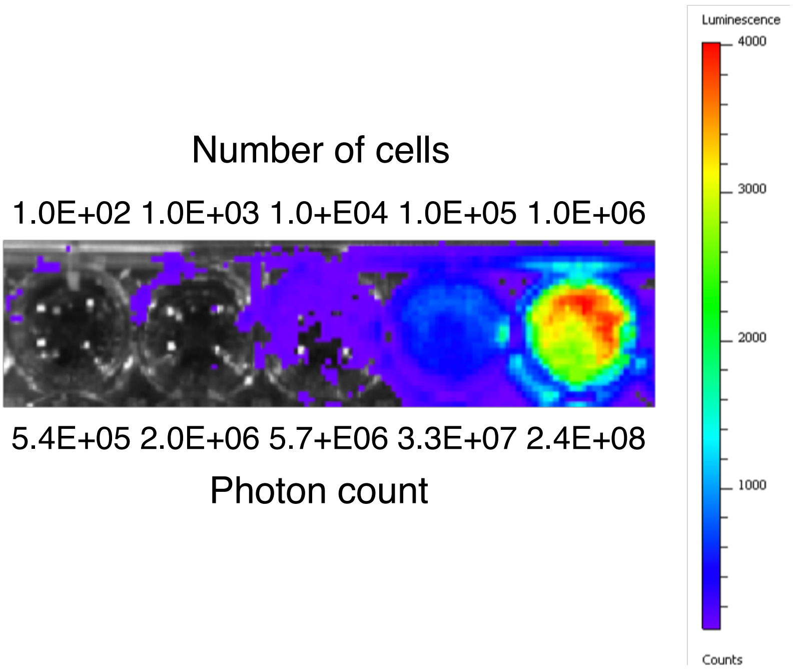
Number of cells
1.0E+02 1.0E+03 1.0+E04 1.0E+05 1.0E+06
5.4E+05 2.0E+06 5.7+E06 3.3E+07 2.4E+08
Photon count
Luminescence
4000
3000
2000
1000
Counts

**B**

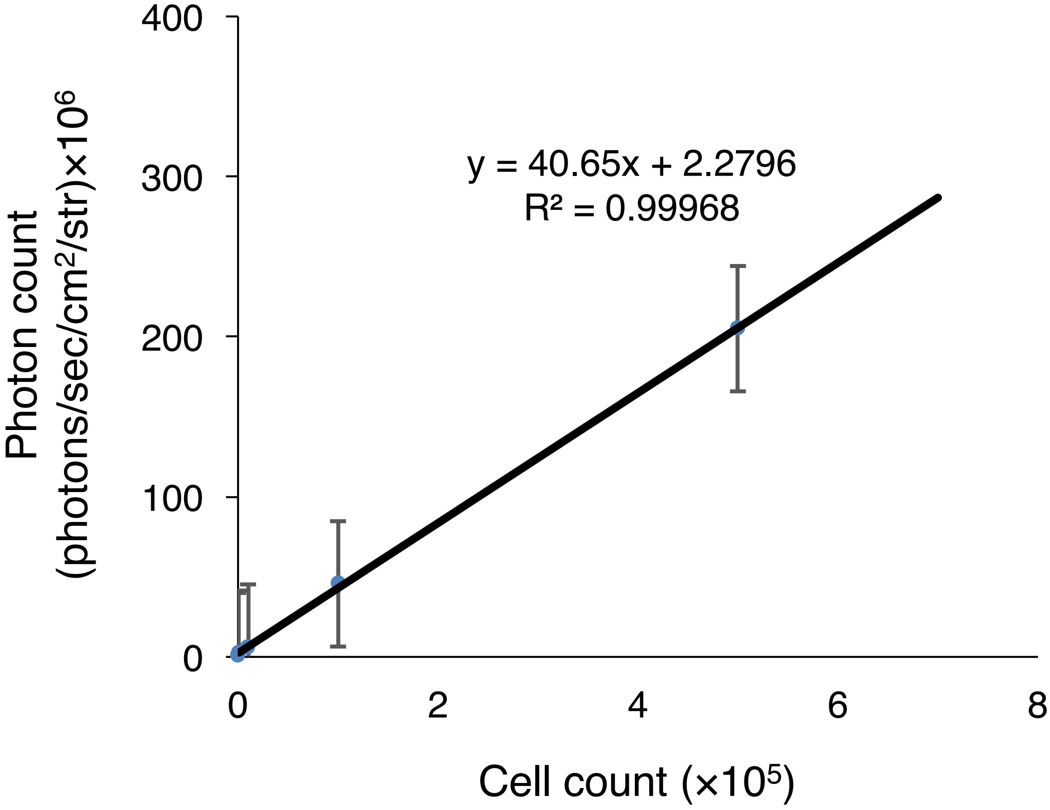
Photon count (photons/sec/cm²/str)×10⁶
400
300
200
100
0
y = 40.65x + 2.2796
R² = 0.99968
0
2
4
6
8
Cell count (×10⁵)

**A**

**B**

**C**

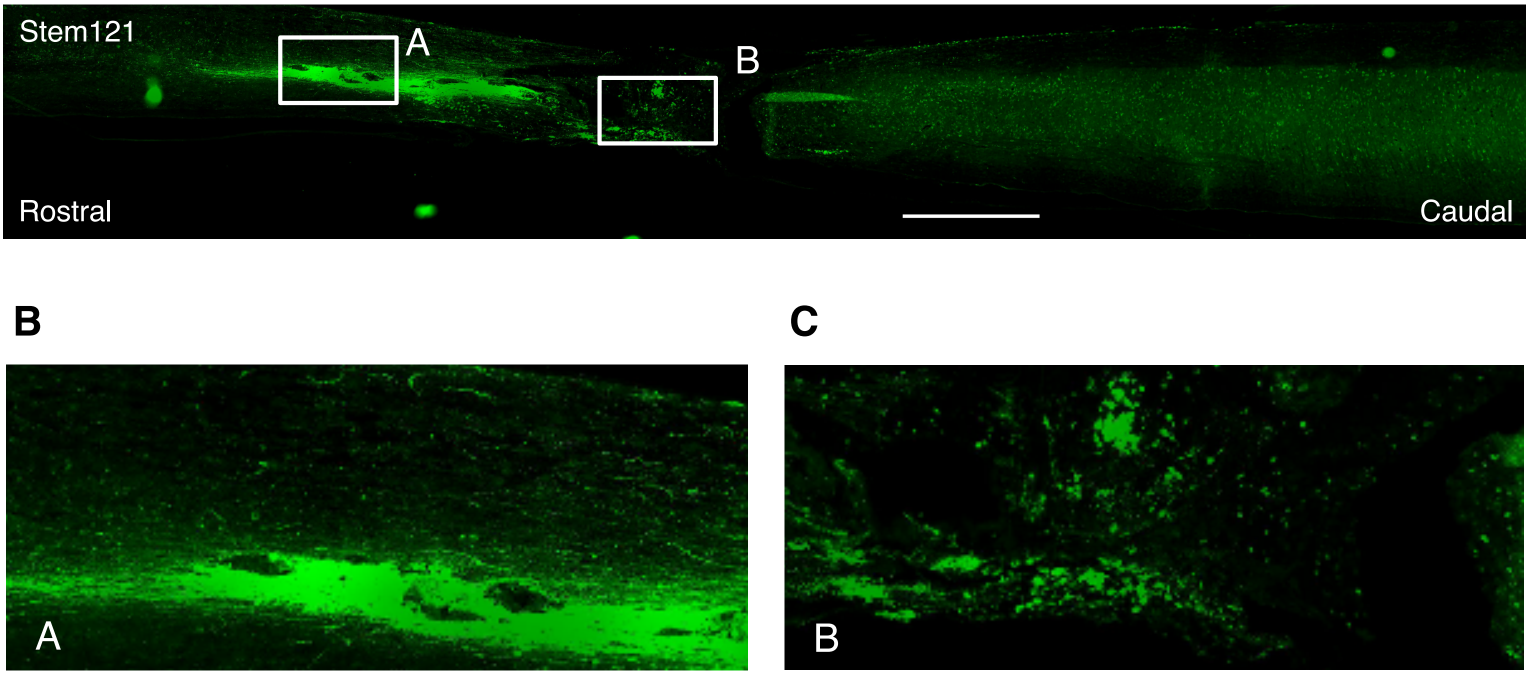

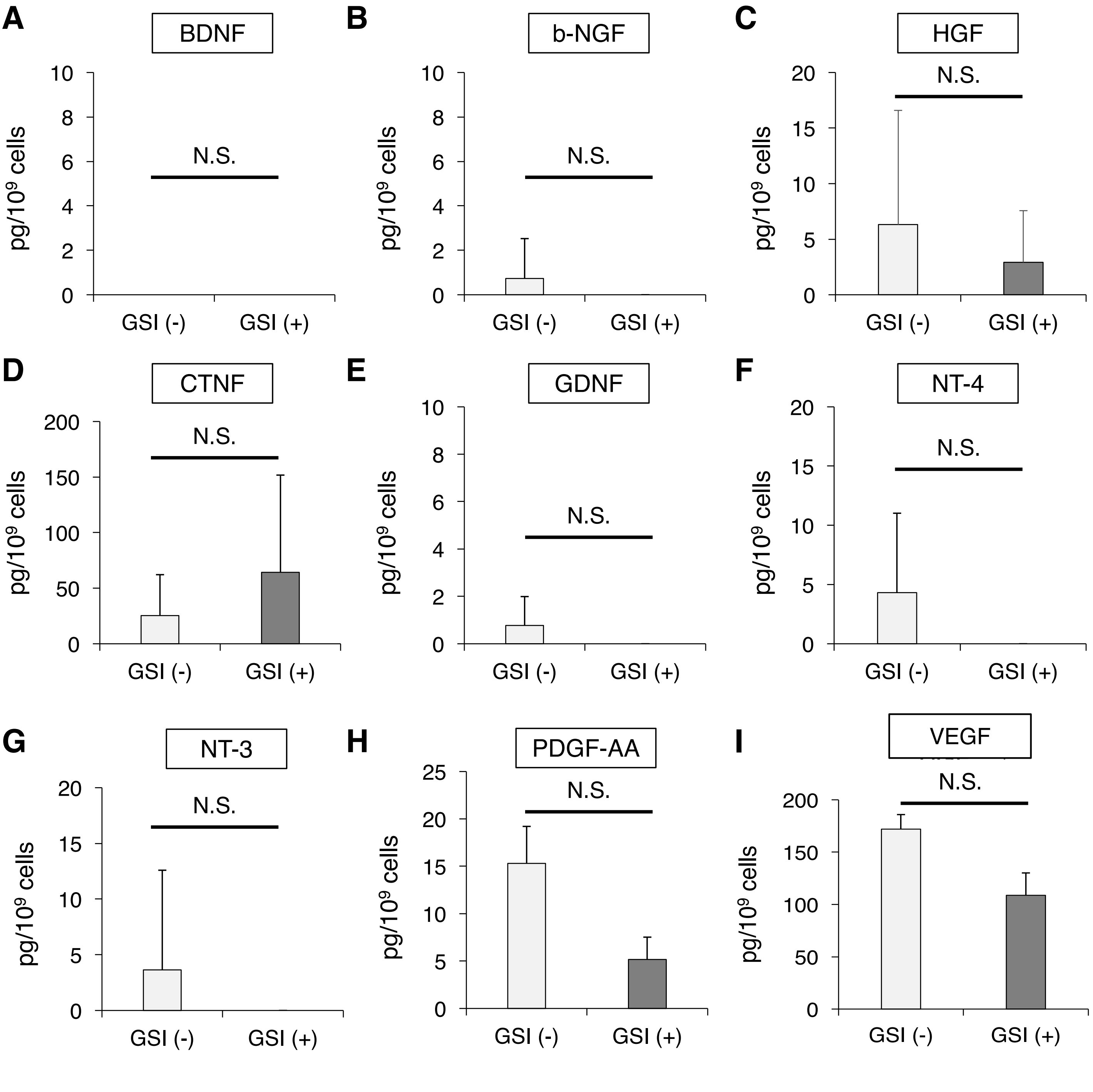

A
BDNF
N.S.
B
b-NGF
N.S.
C
HGF
N.S.
D
CTNF
N.S.
E
GDNF
N.S.
F
NT-4
N.S.
G
NT-3
N.S.
H
PDGF-AA
N.S.
I
VEGF
N.S.
pg/10⁹ cells
GSI (-)
GSI (+)

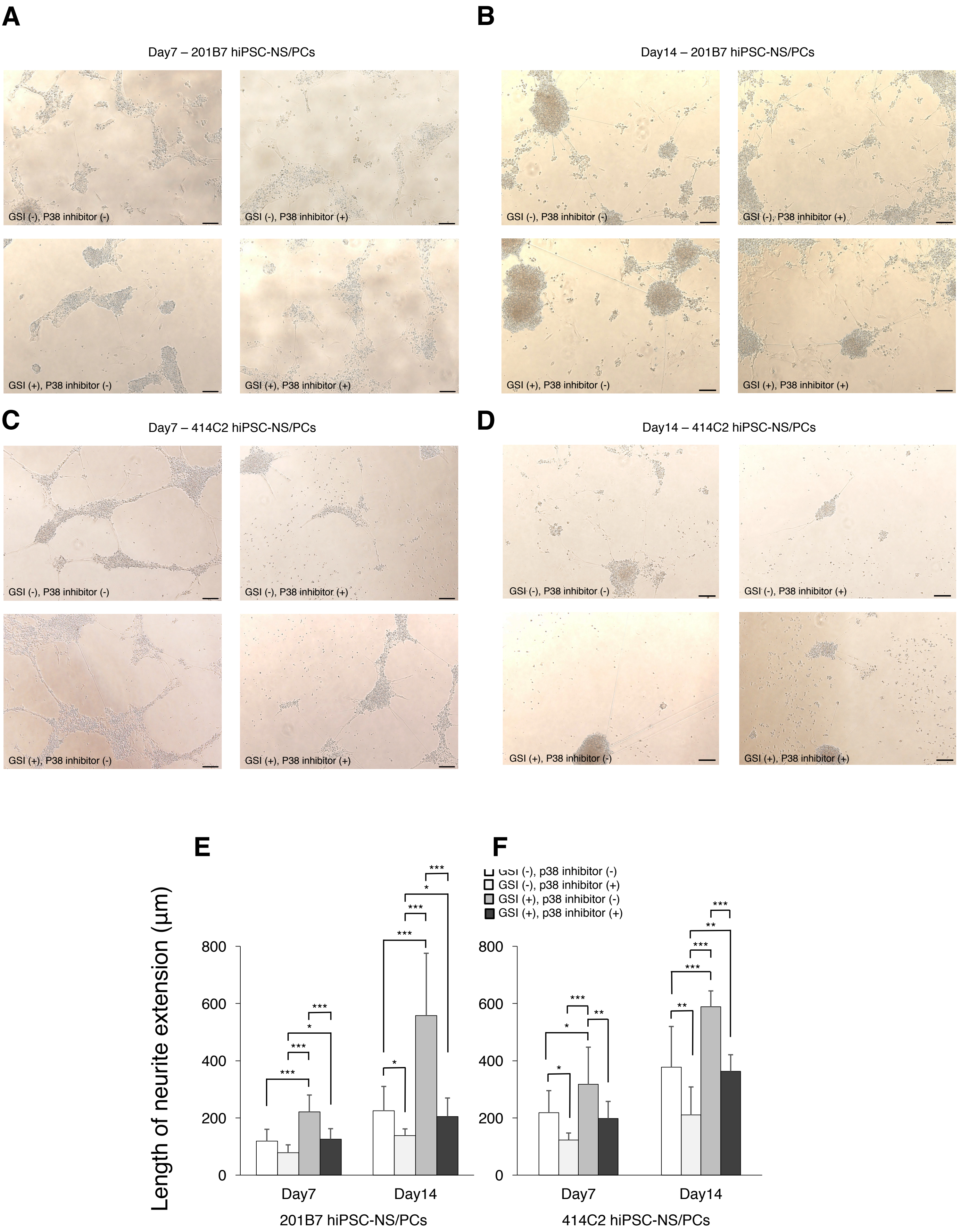
A
Day7 – 201B7 hiPSC-NS/PCs
GSI (-), P38 inhibitor (-)
GSI (-), P38 inhibitor (+)
GSI (+), P38 inhibitor (-)
GSI (+), P38 inhibitor (+)
B
Day14 – 201B7 hiPSC-NS/PCs
GSI (-), P38 inhibitor (-)
GSI (-), P38 inhibitor (+)
GSI (+), P38 inhibitor (-)
GSI (+), P38 inhibitor (+)
C
Day7 – 414C2 hiPSC-NS/PCs
GSI (-), P38 inhibitor (-)
GSI (-), P38 inhibitor (+)
GSI (+), P38 inhibitor (-)
GSI (+), P38 inhibitor (+)
D
Day14 – 414C2 hiPSC-NS/PCs
GSI (-), P38 inhibitor (-)
GSI (-), P38 inhibitor (+)
GSI (+), P38 inhibitor (-)
GSI (+), P38 inhibitor (+)
E
F
GSI (-), p38 inhibitor (-)
GSI (-), p38 inhibitor (+)
GSI (+), p38 inhibitor (-)
GSI (+), p38 inhibitor (+)
Length of neurite extension (µm)
0
200
400
600
800
Day7
Day14
201B7 hiPSC-NS/PCs
414C2 hiPSC-NS/PCs

**Supplemental Figure Legends**

**Figure S1. Detection of Bioluminescence and Fluorescence Signals in Lentivirally Transfected hiPSC-NS/PCs *in vitro***

(A) Bioluminescence imaging was used to detect bioluminescence signals in various numbers of hiPSC-NS/PCs (0, $1\times10^2$, $1\times10^3$, $1\times10^4$, $1\times10^5$, and $5\times10^5$ cells per well).

(B) BLI significantly find out a direct linear correlation between cell numbers and photon counts *in vitro* (n = 5 independent experiments).

The data are presented as the means ± SEM. BLI; bioluminescence imaging

**Figure S2. Grafted cells were extended from rostral to caudal site over the epicenter of chronically injured spinal cord**

(A) In the GSI (+) group, representative immunohistological images of STEM121 positive cells in the chronically injured spinal cord at 84 days after transplantation. Scale bars, 1,000 mm.

(B) and (C) Boxed area in (A).

**Figure S3. Neurotrophic Factors Are Not Secreted into the Culture Medium of GSI-treated hiPSC-NS/PCs**

(A) – (I) Quantification of secreted cytokines from GSI-treated hiPSC-NS/PCs and conventional hiPSC-NS/PCs. GSI-treated hiPSC-NS/PCs showed no significant difference in the secretion of various neurotrophic factors into the culture medium compared with conventional hiPSC-NS/PCs according to the Wilcoxon rank-sum test (n = 3 independent experiments). N.S. = Non-significant. The data are presented as the mean ± SEM.

**Figure S4. Quantification of Neurite Extension from hiPSC-NS/PCs Treated with GSI Alone, a p38 MAPK Inhibitor Alone or a Combination of Both**

(A) - (D) Micrographs showing representative neurite outgrowth from hiPSC-NS/PCs treated with GSI alone, a p38 MAPK inhibitor alone or both at 7 and 14 days after neuronal differentiation. Scale bars, 100 μm.

(E) and (F) Length of neurite extension from each cell measured in 10 separate fields using a microscope and compared among each group (n = 5 independent experiments).

$^{*}p < 0.05$, $^{**}p < 0.01$ and $^{***}p < 0.001$ according to a one-way ANOVA with the Tukey-Kramer test.

**Supplemental Experimental Procedures**

**Treatment of hiPSC-NS/PCs with GSI**

The small molecule GSI, N-[N-(3,5-difluorophenacetyl)-l-alanyl]-S-phenylglycine t-butyl ester (DAPT; Sigma-Aldrich, St. Louis, MO, USA, D5942), is a potent nontransition-state analog inhibitor of g-secretase that is thought to interact with the same active site between presenilin-1 heterodimers in gamma-secretase complexes. DAPT was dissolved in DMSO at a final concentration of 10 mM. The highest concentration of this molecule that effectively inhibited the division of hiPSC-NS/PCs without precipitating in culture medium or producing toxic effects was determined in preliminary experiments. For cell transplantation assays, the hiPSC-NS/PCs were used after the fifth passage.

**Histological analyses**

Animals were anesthetized and transcardially euthanized with 0.1 M PBS containing 4% paraformaldehyde

(PFA) at 84 days after transplantation. Their spinal cords were then removed, postfixed overnight in 4% PFA, soaked overnight in 10% sucrose, followed by 30% sucrose, embedded in Optimal Cutting Temperature (O.C.T) compound (Sakura Finetechnical Co., Ltd., Tokyo, Japan), frozen, and sectioned along the sagittal and axial planes at a 14 μm thickness on a cryostat (CM3050S, Leica Microsystems, Wetzlar, Germany). Spinal cord sections were histologically evaluated by staining with HE and LFB and through immunohistochemistry (IHC). Tissue sections were stained with the following primary antibodies for IHC: anti-pan-ELAVL (Hu) (human IgG, 1:1000, a gift from Dr. Robert Darnell; The Rockefeller University, New York, NY, USA), anti-GFAP (rabbit IgG, 1:200; Dako, Carpinteria, CA, USA, Z0334), anti-APC CC-1 (mouse IgG, 1:300; Abcam, Cambridge, UK, ab16794), anti-human-specific Nestin protein (rabbit IgG, 1:300; described previously (Kanemura et al., 2002)), anti-Ki67 (rabbit IgG, 1:200; Leica Biosystems, Wetzlar, Germany), anti-human nuclear antigen (anti-HNA, mouse IgG, 1:200; Chemicon, Temecula, CA, USA, MAB1281), anti-neurofilament 200 kDa (NF-H, mouse IgG, 1:200; Merck Millipore, Billerica, MA, USA, MAB5266), anti-5-hydroxytrypamine (5HT; goat IgG, 1:200; Immunostar, Hudson, WI, USA, 20080), anti-p38 MAPK (p38; rabbit IgG, 1:200; Cell Signaling Technology, Danvers, MA, USA, 8690L), anti-phospho-p38 MAPK (Pp38; rabbit IgG, 1:200; Cell Signaling Technology, Danvers, MA, USA, 4511L), anti-GAD67 (mouse IgG2a, 1:200; Merck Millipore, Billerica, MA,

USA, MAB5406), anti-VGLuT1 (rabbit IgG, 1:100; Abcam, Cambridge, UK, ab72311), anti-human cytoplasm (STEM121; mouse IgG1, 1:300; Takara Bio, Kusatsu, Japan, Y40410), anti-β-tubulin (Tuj1; mouse IgG, 1:300; Sigma-Aldrich, St. Louis, MO, USA, T8660), anti-Bsn (mouse IgG, 1:200; Stressgen, ADI-VAM-PS003), anti-hSyn (Mouse IgG, 1:200; Chemicon, Temecula, CA, USA, MAB332), anti-PSD95 (rabbit, 1:100; Cell Signaling Technology, Danvers, MA, USA, 2507), and anti-Gephyrin (rabbit IgG, 1:250; Synaptic Systems, Göttingen, Germany, 147008). Nuclei were stained with Hoechst 33258 (10 µg/ml, Sigma-Aldrich, St. Louis, MO, USA). For IHC of RtST fibers, a streptavidin secondary antibody (Alexa Fluor 555 conjugate, 1:500; Thermo Fisher, Waltham, MA, USA, S32355) was used. Samples were visualized using a fluorescence microscope (BZ-X710; Keyence Co., Osaka, Japan) or a confocal laser-scanning microscope (LSM 700, Carl Zeiss, Jena, Germany). The numbers of marker-positive cells, such as HNA-, Ki67-, Nestin-, pan-ELAVL (Hu)-, GFAP-, and APC-positive cells, as well as NF-H-, 5-HT-, and BDA-labeled RtST-positive fibers were counted in each section (n = 5 per group).
