# Supplementary material for: Treatment with a Gamma-Secretase Inhibitor Promotes Functional Recovery in Human iPSC- Derived Transplants for Chronic Spinal Cord Injury
Source: Stem Cell Reports. 2018 Nov 29;11(6):1416–32. doi: 10.1016/j.stemcr.2018.10.022 (PMC6294244; doi:10.1016/j.stemcr.2018.10.022)
Supplement: Document S2. Article plus Supplemental Information [file mmc2.pdf]

Stem Cell Reports
Article

OPEN ACCESS

Treatment with a Gamma-Secretase Inhibitor Promotes Functional Recovery in Human iPSC- Derived Transplants for Chronic Spinal Cord Injury

Toshiki Okubo,[1,2] Narihito Nagoshi,[1] Jun Kohyama,[2] Osahiko Tsuji,[1] Munehisa Shinozaki,[2] Shinsuke Shibata,[2,3] Yoshitaka Kase,[2,4] Morio Matsumoto,[1] Masaya Nakamura,[1,*] and Hideyuki Okano[2,3,*]
[1]Department of Orthopaedic Surgery, Keio University School of Medicine, 35 Shinanomachi, Shinjuku-ku, Tokyo 160-8582, Japan
[2]Department of Physiology, Keio University School of Medicine, 35 Shinanomachi, Shinjuku-ku, Tokyo 160-8582, Japan
[3]Electron Microscope Laboratory, Keio University School of Medicine, 35 Shinanomachi, Shinjuku-ku, Tokyo 160-8582, Japan
[4]Department of Geriatric Medicine, Graduate School of Medicine, University of Tokyo, Bunkyo-ku, Tokyo 113-8655, Japan
*Correspondence: masa@a8.keio.jp (M.N.), hidokano@a2.keio.jp (H.O.)
https://doi.org/10.1016/j.stemcr.2018.10.022

SUMMARY

Treatment involving regenerative medicine for chronic spinal cord injury (SCI) is difficult due to phase-dependent changes in the intraspinal environment. We previously reported that treatment with a gamma-secretase inhibitor (GSI), which inhibits Notch signaling, promotes the differentiation into mature neurons in human induced pluripotent stem cell-derived neural stem/progenitor cell (hiPSC-NS/PC) transplantation for subacute SCI. Here, we evaluated the efficacy of GSI-treated hiPSC-NS/PC transplantation in treating chronic SCI, which resulted in significantly enhanced axonal regrowth, remyelination, inhibitory synapse formation with the host neural circuitry, and reticulo spinal tract fiber formation. Interestingly, inhibiting Notch signaling with GSI caused phosphorylation of p38 MAPK, which is a key molecule required to promote axonal regeneration. These favorable outcomes contributed to motor function improvement. Therefore, treating cells with GSI provides a beneficial effect after transplantation, even in the chronic phase following SCI.

INTRODUCTION

Spinal cord injury (SCI) is a catastrophic trauma that causes permanent paralysis, sensory disturbances, neuropathic pain, and bowel-bladder dysfunction (Mothe and Tator, 2013). Demographically, most patients with SCI are in the chronic phase, and therapeutic development becomes imperative in this stage. Numerous studies have demonstrated the efficacy of transplanting neural stem/progenitor cells (NS/PCs) for the treatment of SCI, but the optimal time window for transplantation is regarded as the subacute phase after injury (Cummings et al., 2005; Nori et al., 2011; Okano et al., 2013; Salewski et al., 2015). Treatment of chronic SCI is difficult due to phase-dependent changes in the intramedullary environment, such as glial scar and cavity formation. Unfortunately, reports showing favorable outcomes of targeting the chronic phase have been limited. In fact, several studies have failed to identify significant motor function recovery after cell transplantation in chronically injured spinal cords (Keirstead et al., 2005; Kumamaru et al., 2013; Nishimura et al., 2013). Only a few reports have shed light on this challenging situation and have shown that neural precursor cell transplantation combined with chondroitinase ABC treatment, which promotes the degradation of chondroitin sulfate proteoglycans, improves locomotor function recovery in chronic SCI (Karimi-Abdolrezaee et al., 2010; Suzuki et al., 2017). However, these procedures are technically demanding and are not clinically relevant, due to the necessity for implantation of an intrathecal catheter prior to cell transplantation.

We previously reported that NS/PCs derived from human induced pluripotent stem cells (hiPSC-NS/PCs) treated with a gamma-secretase inhibitor (GSI) (Notch signal inhibitor) exhibit a reduced proportion of dividing cells and increased neuronal maturation *in vitro*. Transplantation of these cells leads to robust axonal regrowth and promotes motor function recovery in rodents with subacute SCI (Okubo et al., 2016). These results prompted the use of GSI treatment for chronic SCI because graft cell-derived neuronal maturation plays beneficial roles in functional improvement by reconstructing neural circuits in the injured spinal cord, even when transplantation is performed in the chronic stage (Suzuki et al., 2017; Tashiro et al., 2016). If GSI-treated NS/PCs exert their ability to extend neuronal axons against the harsh chronic environment and integrate with host tissue through functional synapses, significant effects that could lead to locomotor recovery in the case of transplantation are expected.

Therefore, the purpose of the present study was to evaluate the efficacy of GSI-treated hiPSC-NS/PC transplantation in the chronic phase of SCI. Because GSI treatment takes only 1 day to achieve neuronal induction, this methodology has the potential to become an efficient and useful tool for cell transplantation therapy in SCI.

This is an open access article under the CC BY license (http://creativecommons.org/licenses/by/4.0/).

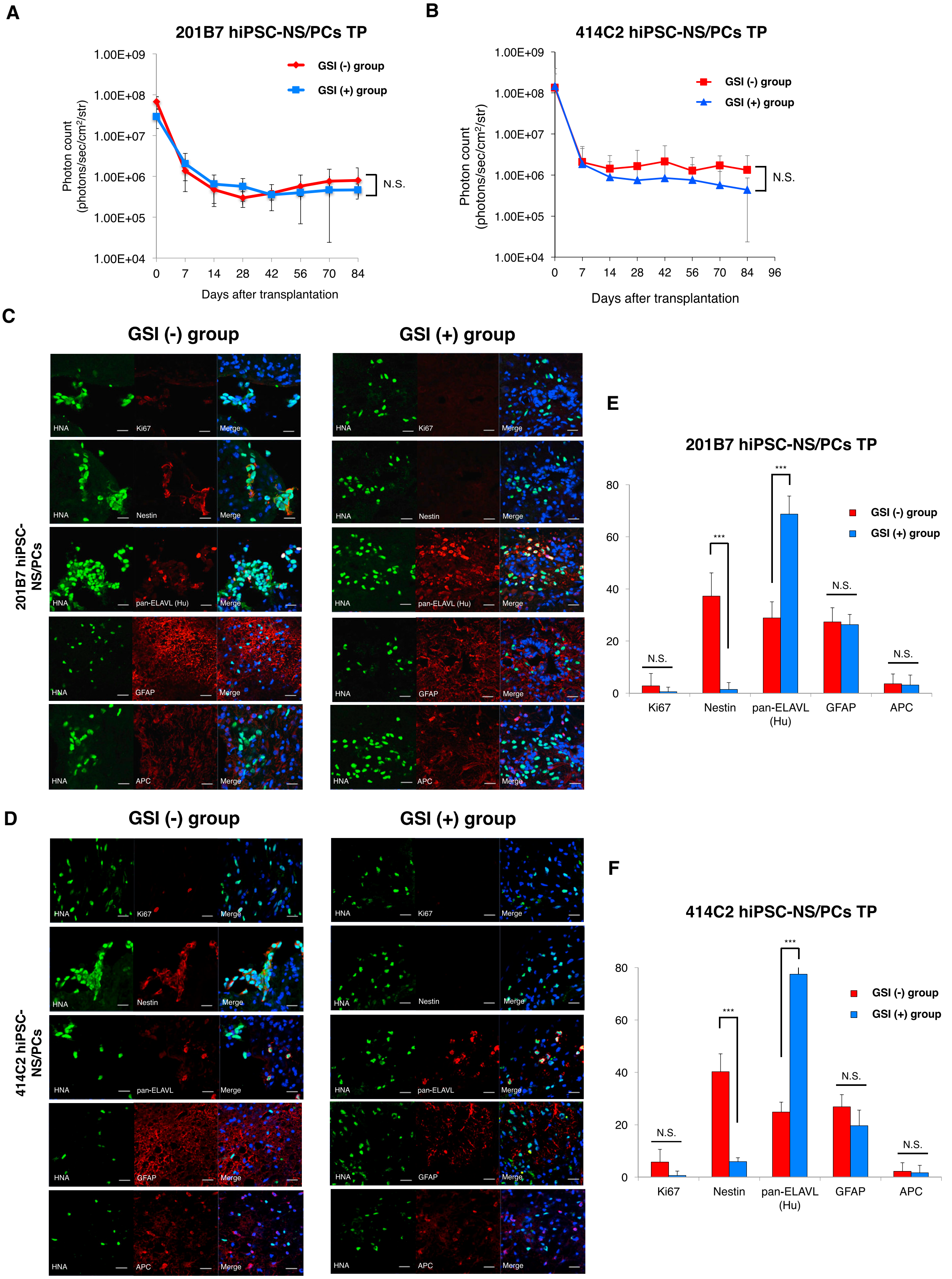

(legend on next page)

RESULTS

GSI-Treated hiPSC-NS/PCs Survive and Differentiate into Mature Neurons Following Transplantation in the Chronic Phase of SCI

Contusive moderate SCI was induced at the Th10 level in spinal cords of adult female non-obese diabetic (NOD)-severe combined immunodeficiency (SCID) (NOD/ShiJic-*scid*Jcl; mice) using an IH impactor (a force defined impact [60 kdyn]) with a stainless steel-tipped impactor. Non-tumorigenic hiPSC-NS/PCs (two cell lines; 201B7 and 414C2) (Nori et al., 2015) treated with or without the small-molecule GSI, N-[N-(3,5-difluorophenacetyl)-l-alanyl]-S-phenylglycine t-butyl ester, were transplanted 42 days after SCI. To monitor the survival and growth of the transplanted cells in the injured mouse spinal cord, hiPSC-NS/PCs were lentivirally transduced with ffLuc, a fusion protein between Venus fluorescent protein and firefly luciferase (Hara-Miyauchi et al., 2012), which allowed transplanted cells to be identified by bioluminescent luciferase signals and fluorescent Venus signals. The sequential evaluation of transplanted cell viability is possible with these bioluminescence imaging (BLI) systems since the luciferin-luciferase reaction depends on ATP and only living cells release photons (Okada, 2005). The photon counts and the number of the transplanted hiPSC-NS/PCs were significantly correlated ($r^2 = 0.999$, Figures S1A–S1C). The photon counts of the transplanted hiPSC-NS/PCs with or without GSI treatment decreased within the first 7 days after transplantation, reached a plateau at day 14, and were maintained until 3 months (Figures 1A and 1B). These results indicated that both GSI-treated and untreated hiPSC-NS/PCs survive following transplantation, even in the chronic phase, without any obvious tumorigenicity.

To evaluate the differentiation phenotype of the transplanted cells at lesion epicenter, immunostaining for various cell markers was examined at 84 days after transplantation and subjected to quantitative analyses. The transplanted hiPSC-NS/PCs differentiated into the three following neural lineages in each group: pan-ELAVL $(Hu)^+$ mature neurons, $GFAP^+$ astrocytes, and APC^+ oligodendrocytes (Figures 1C and 1D). In the GSI (+) group, the proportion of pan-ELAVL $(Hu)^+$ cells increased significantly, but the proportion of $Nestin^+$ cells decreased (Figures 1E and 1F). The proportions of $GFAP^+$ and APC^+ cells were not significantly different between the groups.

Transplantation of hiPSC-NS/PCs with GSI Treatment Prevents Atrophy of the Injured Spinal Cord and Leads to Remyelination

To investigate the cross-sectional and myelinated-positive area of the spinal cord, H&E and Luxol fast blue (LFB) staining were performed at 84 days after transplantation. Representative results of H&E staining of the transverse area of spinal cords are shown in Figures 2A and 2B. Quantitative analyses revealed that the transverse area of the spinal cord at the lesion epicenter and +4 mm caudal area sites was significantly larger in the GSI (+) group than in the GSI (−) and PBS groups (Figures 2C and 2D).

Representative results of LFB staining are shown in Figures 2E and 2F. Quantitative analyses revealed that the GSI (+) group exhibited significantly larger LFB-positive myelinated areas from the epicenter to +1.0 mm caudal sites than the GSI (−) and PBS groups (Figures 2G and 2H). These results indicated that GSI-treated hiPSC-NS/PCs led to remyelination of the chronically injured spinal cord.

GSI-Treated hiPSC-NS/PCs Contribute to Axonal Regrowth after Transplantation in the Chronic Phase of SCI

The effects of transplanted GSI-treated hiPSC-NS/PCs on axonal regrowth after SCI were examined through immunostaining analyses. In the GSI (+) group, human cytoplasmic marker (STEM121)-positive (grafted) cells were extended from rostral to caudal site over the epicenter of chronically injured spinal cord (Figures S2A–S2C). An increased abundance of neurofilament 200 kDa $(NF\text{-}H)^+$ neuronal fibers at lesion epicenter and +4 mm rostral or caudal from injury site (Figures 3A–3D) and 5-hydroxytrytamine

Figure 1. Long-Term Observation after Transplantation of GSI-Treated hiPSC-NS/PCs in the Chronic Phase of SCI

(A and B) Quantitative analyses of photon counts derived from transplanted cells at 84 days post transplantation: (A) 201B7 cell line; PBS group, n = 10; GSI (−) group, n = 10; GSI (+) group, n = 10 mice; and (B) 414C2 cell line; PBS group, n = 10; GSI (−) group, n = 10; GSI (+) group, n = 10 mice.

(C and D) Representative images of immunohistochemical staining for each group at the injury site. HNA^+ transplanted cells were stained with Ki67, Nestin, pan-ELAVL (Hu), GFAP, and APC. Blue signals show Hoechst-positive cells. (C) 201B7 cell line and (D) 414C2 cell line. Scale bars, 20 μm.

(E and F) Percentages of cell-type-specific, marker-positive cells among the HNA^+ transplanted cells at 84 days after transplantation: (E) 201B7 cell line; PBS group, n = 10; GSI (−) group, n = 10; GSI (+) group, n = 10 mice; and (F) 414C2 cell line; PBS group, n = 10; GSI (−) group, n = 10; GSI (+) group, n = 10 mice.

***$p < 0.001$ and N.S., non-significant according to a Wilcoxon rank-sum test (A, B, E, and F). The data are presented as the means ± SEM.

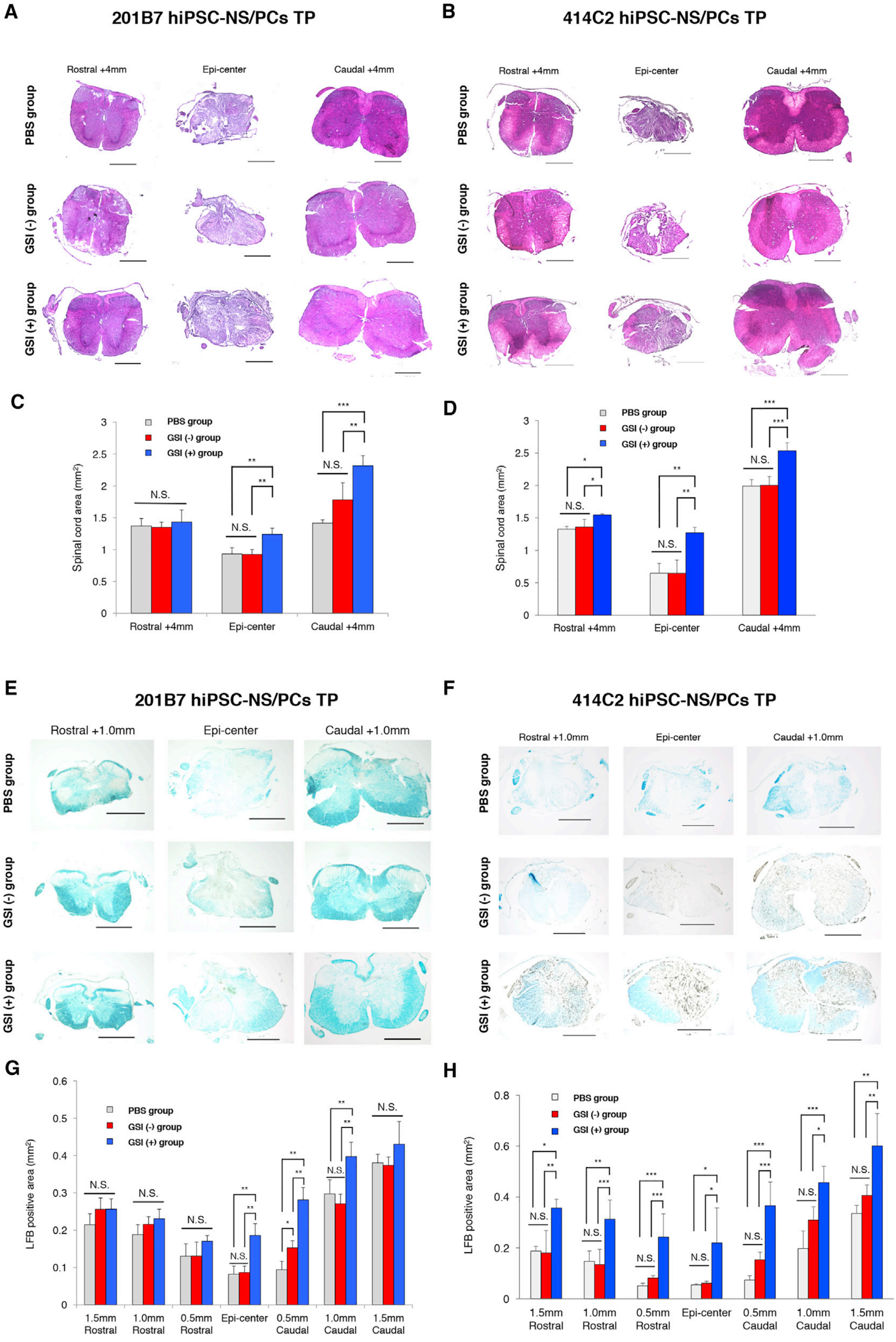

(legend on next page)

(5HT)$^+$ raphespinal serotonergic fibers at the lumbar enlargement (Figures 3E–3H) was observed in the GSI (+) group at all sites. Moreover, immunoelectron microscopy analyses revealed that many transplanted cell-derived regenerative axons were actively remyelinated by host-derived glial cells in the chronically injured spinal cord (Figures 3I and 3J).

p38 Phosphorylation Induces Neurite Outgrowth by Inhibiting Notch Signaling

Using a cytokine antibody array, we previously showed that conventional hiPSC-NS/PCs secrete few neurotrophic factors into the culture medium, whereas hiPSC-oligodendrocyte precursor cell-enriched NS/PCs secrete vascular endothelial growth factor (VEGF) and platelet-derived growth factor (PDGF)-AA (Kawabata et al., 2016). Thus, *in vitro* cytokine profiles were analyzed to investigate whether GSI-treated hiPSC-NS/PCs have the potential to supply neurotrophic factors for neurite outgrowth. However, GSI-treated hiPSC-NS/PCs did not secrete significantly greater amounts of various neurotrophic factors into the culture medium than conventional hiPSC-NS/PCs (Figures S3A–S3I).

To elucidate the mechanism of GSI-treated hiPSC-NS/PC-induction of axonal regrowth in the chronically injured spinal cord, we focused on the p38 MAPK pathway. The phosphorylation of p38 MAPK is regulated by Notch signaling through the expression of DUSP1 (a protein phosphatase involved in MAPK regulation), and this pathway plays an important role in axonal regeneration (Kondoh et al., 2007; Nix et al., 2011). To analyze the effects of GSI and a p38 MAPK inhibitor (SB203580) on neurite outgrowth, neural differentiation of hiPSC-NS/PCs was induced *in vitro*, and the length of extended neurons was measured at 7 and 14 days. After treatment of hiPSC-NS/PCs with GSI, the length of neurite outgrowth was significantly greater than in the non-treated group, whereas neurite outgrowth length was significantly reduced after treatment with the p38 MAPK inhibitor (Figures S4A–S4F). Interestingly, after treatment with both GSI and the p38 MAPK inhibitor, the length of neurite outgrowth was almost comparable with that in the non-treated group. Immunostaining for various cell markers was performed at 14 days after neural induction, and representative results are shown in Figures 4A and 4B. In the GSI treatment group, βIII-tubulin$^+$ neurons and the p38-positive area co-localized, and a phosphorylated p38-positive area was identified in the nucleus. The relative amount of unphosphorylated p38 protein was not significantly different between the GSI (−) and GSI (+) groups, but phosphorylated p38 was upregulated in the GSI (+) group, as shown by western blot analysis (Figures 4C–4E). Furthermore, immunostaining for various cell markers was performed at 84 days after cell transplantation for chronic phase injured spinal cord, and representative results are shown in Figure 4F. In the GSI treatment group, STEM121$^+$ transplanted cells and the phosphorylated p38-positive area co-localized. Percentages of the phosphorylated p38-positive cells among the STEM121$^+$ transplanted cells were significantly increased compared with the GSI (−) group (Figure 4G). Taken together, these results suggested that GSI treatment promotes the phosphorylation of p38 MAPK, leading to axonal regrowth.

hiPSC-NS/PC-Derived Mature Neurons Are GABAergic and Integrate with the Host Neural Circuitry as an Inhibitory Synapse

Interventions in serotonergic activity recover locomotor function after SCI through activation of the central pattern generator (CPG) (Ghosh and Pearse, 2014). To assess the efficacy of GSI-treated hiPSC-NS/PC transplantation for the CPG, the immunostaining of neurotransmitters related to both the excitatory and inhibitory control of the CPG was examined using specific markers. Few STEM121$^+$/vesicular glutamate transporter-1$^+$ excitatory neurons were observed. In contrast, quantitative analysis revealed that 78% of STEM121$^+$ cells were positive for glutamic acid decarboxylase 67 (GAD67) inhibitory neurons, indicating

Figure 2. Transplantation of GSI-Treated hiPSC-NS/PCs Prevents Atrophy of the Injured Spinal Cord and Leads to Remyelination
(A and B) Representative H&E-stained images of axial sections at the lesion epicenter and at sites located 4 mm rostral and caudal in each group. (A) 201B7 cell line and (B) 414C2 cell line. Scale bars, 200 μm.
(C and D) Quantitative analyses of spinal cord area measured in H&E-stained axial sections in different regions: (C) 201B7 cell line; PBS group, n = 10; GSI (−) group, n = 10; GSI (+) group, n = 10 mice; and (D) 414C2 cell line; PBS group, n = 10; GSI (−) group, n = 10; GSI (+) group, n = 10 mice.
(E and F) Representative LFB-stained images of axial sections at the lesion epicenter and at sites located 1.5 mm rostral and caudal. (E) 201B7 cell line and (F) 414C2 cell line. Scale bars, 200 μm.
(G and H) Quantitative analyses of spinal cord area measured in LFB-stained axial sections in different regions: (G) 201B7 cell line; PBS group, n = 10; GSI (−) group, n = 10; GSI (+) group, n = 10 mice; and (H) 414C2 cell line; PBS group, n = 10; GSI (−) group, n = 10; GSI (+) group, n = 10 mice.
*p < 0.05, **p < 0.01, ***p < 0.001, and N.S., non-significant according to one-way ANOVA with the Tukey-Kramer test (C, D, G, and H). The data are presented as the means ± SEM.

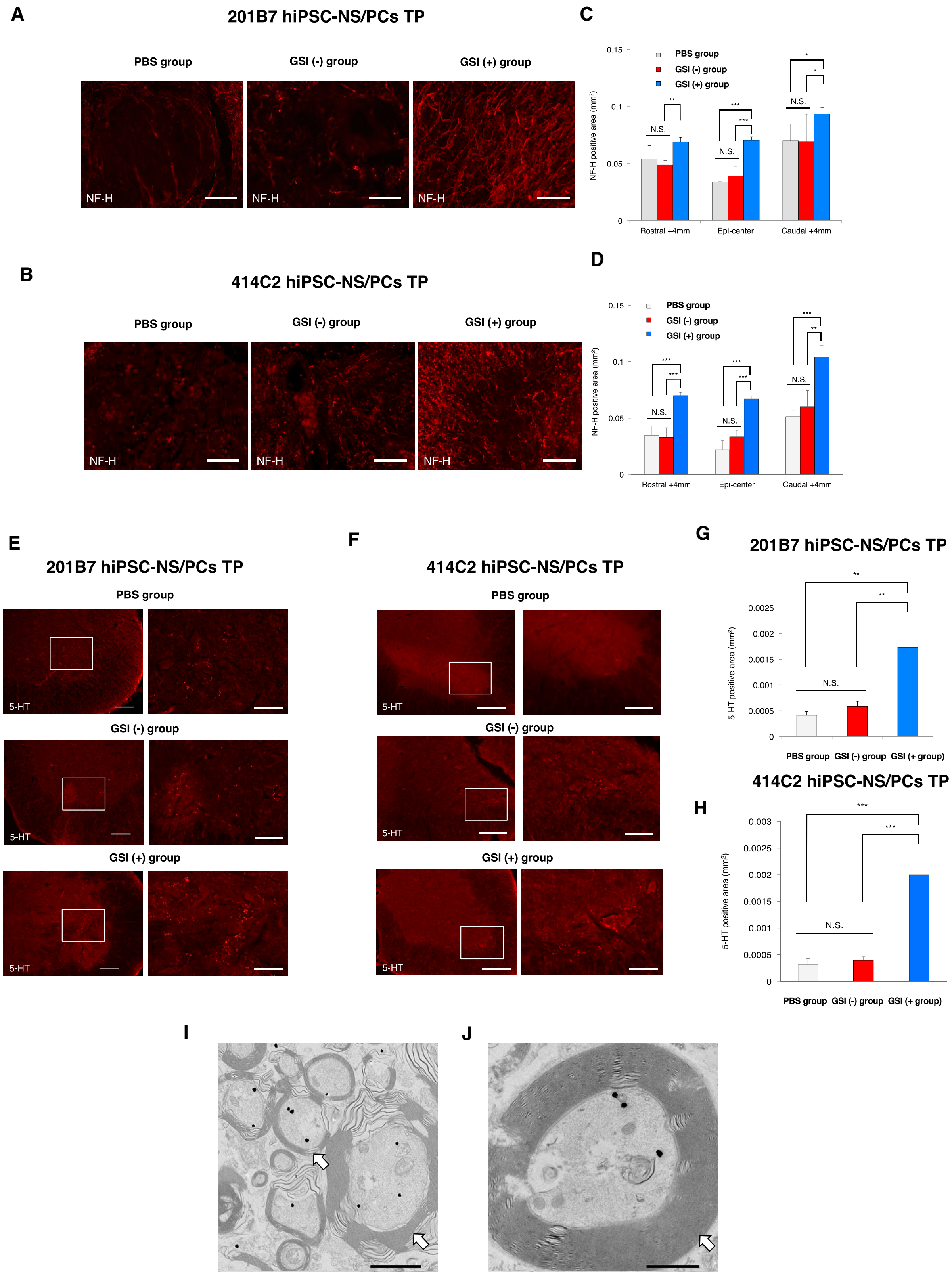

(legend on next page)

that the transplanted cell-derived neurons were GABAergic (Figure 5A). The STEM121$^+$ transplanted cells also connected with STEM121$^-$/GAD67$^+$ host mouse cells (Figure 5B). To determine the ability of GSI-treated transplanted cell-derived neurons to integrate with the host neural circuitry, the immunostaining of various synaptic markers was examined. βIII-tubulin$^+$/human nuclear antigen (HNA)$^+$ cells transplanted in parenchymal locations co-localized with boutons of the host neurons positive for the mouse-specific presynaptic marker Bassoon (Figure 5C), and boutons positive for the human-specific presynaptic marker synaptophysin apposed the host mouse neurons (βIII-tubulin$^+$/HNA$^-$) (Figure 5D). Moreover, boutons positive for postsynaptic density protein 95, a postsynaptic marker of the boutons of excitatory synapses, were very rare, while boutons positive for Gephryin$^+$, a postsynaptic marker of the boutons of inhibitory synapses, apposed the transplanted cells (STEM121$^+$) (Figures 5E and 5F). Immunoelectron microscopy analyses revealed that there was a large number of STEM121$^+$ (i.e., human) presynaptic and postsynaptic structures, and synaptic connections were observed between STEM121$^+$ transplanted cell-derived neurons and host mouse neurons at the injured spinal cord site (Figures 5G and 5H).

Transplantation of GSI-Treated hiPSC-NS/PCs Increases the Reticulo Spinal Tract

The reticulo spinal tract (RtST) descends from the reticular formation and terminates in the spinal cord, which plays a predominant role in the initiation of locomotion and postural control (May et al., 2017; Garcia-Alias et al., 2015). To evaluate RtST fiber regeneration from the brain stem, biotin dextran amine (BDA)-mediated mono-synaptic neuronal fiber tracing was performed at 77 days after transplantation. In the GSI (+) group, the BDA-labeled RtST fibers extending from the rostral side of the spinal cord ran toward the caudal side over the lesion epicenter (Figures 6A and 6B). Furthermore, these fibers were also apparent in the caudal area, and their standardized proportion was significantly increased at the epicenter and +3 mm caudal area (Figure 6C).

Transplantation of GSI-Treated hiPSC-NS/PCs Promotes and Maintains Motor Function Recovery in Chronic SCI

Finally, the Basso Mouse Scale (BMS) score (Basso et al., 2006), rotarod testing (Ito et al., 2009; Okada et al., 2004), and treadmill gait analyses using the DigiGait system were utilized to evaluate locomotor function after transplantation (Li et al., 2005; Springer et al., 2010). In the GSI (−) group, there were no significant improvements in functional recovery compared with the PBS group. In contrast, in the GSI (+) group, significantly greater functional recovery was observed at 56 days after transplantation, and the gained function was maintained thereafter (Figures 7A and 7B). The gait performance of the mice in each group was analyzed using the rotarod test and the DigiGait system at 84 days after transplantation. In the GSI (+) group, treadmill gait analyses revealed a significantly longer stride length and smaller stance angle, and all of the mice walked sufficiently well on a treadmill at 7 cm/s to perform the test (Figures 7C–7F). Moreover, the mice remained on the rotating rod for a significantly longer time in the GSI (+) group (Figures 7G and 7H). The BMS scores and the BDA-labeled RtST standardized portion were significantly correlated. In particular, the correlation coefficient was highest at +3 mm caudal from the epicenter (R^2 = 0.855, Figure 7I).

Figure 3. GSI-Treated hiPSC-NS/PCs Contribute to Axonal Regrowth after Transplantation in the Chronic Phase of SCI

(A and B) Representative images of immunohistochemical staining for NF-H at the lesion epicenter and +4 mm rostral or caudal from injury site in each group. (A) 201B7 cell line and (B) 414C2 cell line. Scale bars, 200 μm.

(C and D) Quantitative analyses of the NF-H-positive area at the lesion epicenter and +4 mm rostral or caudal from injury site in each group (C) 201B7 cell line; PBS group, n = 10; GSI (−) group, n = 10; GSI (+) group, n = 10 mice and (D) 414C2 cell line; PBS group, n = 10; GSI (−) group, n = 10; GSI (+) group, n = 10 mice.

(E and F) Representative images of immunohistochemical staining for 5-HT at the lumbar intumescence for each group. (E) 201B7 cell line and (F) 414C2 cell line. Scale bars, 200 μm.

(G and H) Quantitative analyses of the 5-HT-positive area at the lumbar intumescence in each group (G) 201B7 cell line; PBS group, n = 10; GSI (−) group, n = 10; GSI (+) group, n = 10 mice and (H) 414C2 cell line; PBS group, n = 10; GSI (−) group, n = 10; GSI (+) group, n = 10 mice.

(I) Representative immunoelectron microscopy images. Transplanted cells were detected by anti-human cytoplasm antibody (STEM121) staining. GSI-treated human cell-derived axons were labeled with a STEM121 antibody to show enhanced active remyelination in the mouse spinal cord (white arrows).

(J) At a higher magnification, it can be seen that GSI-treated human cell-derived axons were remyelinated with thick myelin lamellae by host mouse cells (white arrow). Scale bars, 2 μm in (I) and 200 nm in (J).

*$p < 0.05$, **$p < 0.01$, ***$p < 0.001$, and N.S., non-significant according to one-way ANOVA with the Tukey-Kramer test (C, D, G, and H). The data are presented as the means ± SEM.

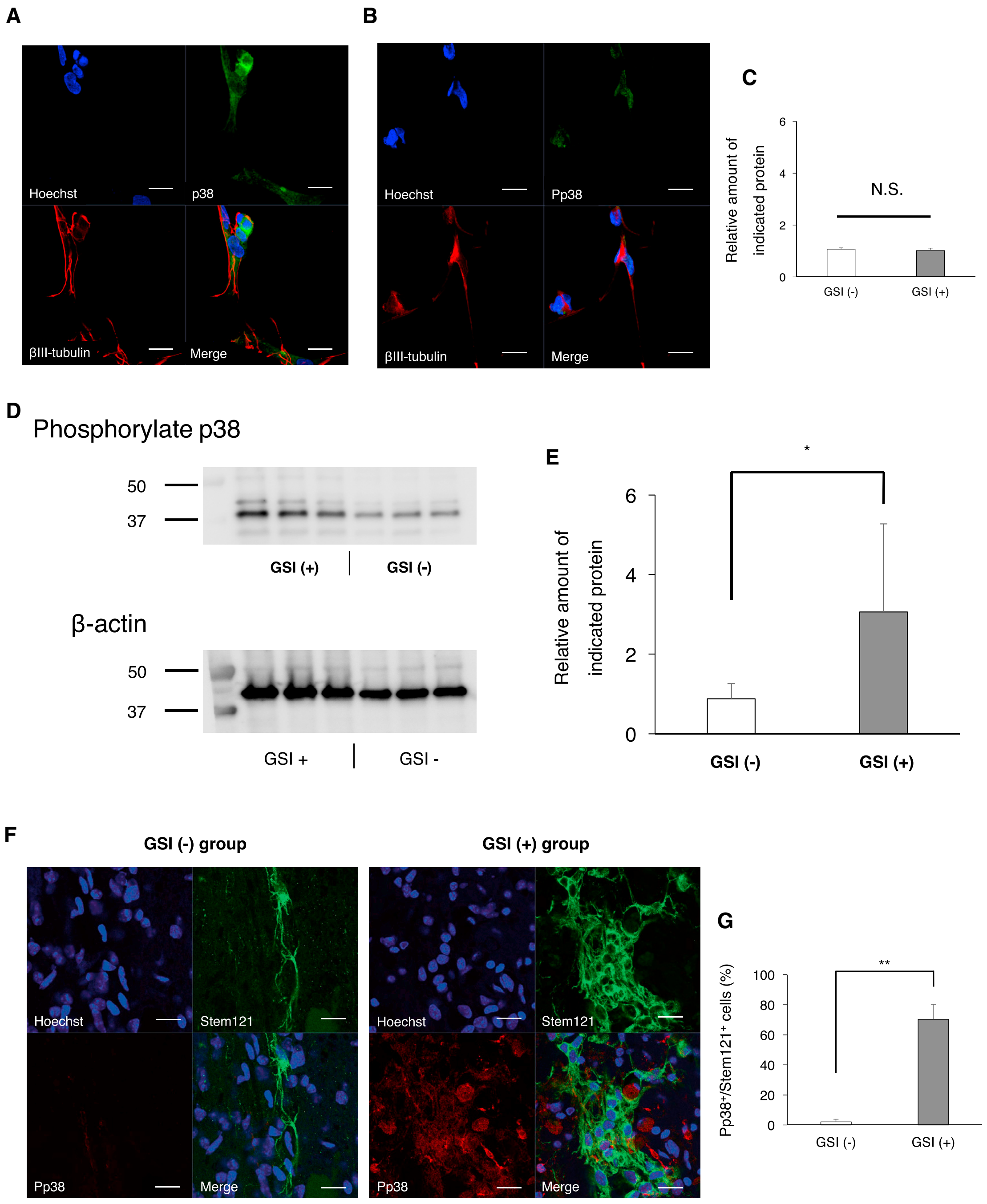

Figure 4. p38 Phosphorylation Induces Neurite Outgrowth by Inhibiting Notch Signaling

(A) Representative *in vitro* images of immunohistochemistry staining for p38 and βIII-tubulin. p38^{+} cells contacted βIII-tubulin^{+} neurons. Scale bars, 20 μm.

(B) Representative images of immunohistochemical staining for phosphorylated p38 and βIII-tubulin. The area of phosphorylated p38^{+} was identified in the nucleus. Scale bars, 20 μm.

(C–E) The protein levels of unphosphorylated and phosphorylated p38 in hiPSC-NS/PCs with or without GSI treatment were analyzed by western blotting. β-actin was assessed as a loading control. Comparison of the protein levels of (C) unphosphorylated and (E) phosphorylated p38 among the PBS, GSI (−), and GSI (+) groups. (D) Representative images of western blotting for phosphorylated p38 and β-actin.

(legend continued on next page)

DISCUSSION

In the present study, we showed that GSI-treated hiPSC-NS/PCs induce regenerative axons, remyelination by host-derived glial cells, and extension of RtST fibers when transplanted into a chronically injured spinal cord. In particular, the mechanism of axonal regrowth involved p38 phosphorylation (i.e., activation), resulting from decreased expression of DUSP1, a protein phosphatase with dual specificity for tyrosine and threonine (Nix et al., 2011), after inhibition of Notch signaling through GSI treatment. These regenerative axons were connected to the host neural network through inhibitory synaptic formation and consequently contributed to significant motor function recovery. Therefore, GSI treatment only 1 day before transplantation has a clinically meaningful effect and shows great potential to enhance spinal cord regeneration, even when transplantation is performed in the chronic phase. However, it is known that other signaling molecules act as targets for GSI (Kopan and Ilagan, 2004). Therefore, we cannot rule out the possibility that the GSI-induced inhibition of signaling pathways other than Notch contributed to axonal regrowth and remyelination.

Several favorable mechanisms have been proposed to explain the efficacy of cell transplantation in SCI. For example, supplying transplant-derived neurons leads to axon regeneration, which has the potential to enhance axonal plasticity and replace lost neurons to reconstruct host neural circuitry (Barnabe-Heider and Frisen, 2008). Transplanted cell-derived oligodendrocytes are responsible for remyelination of demyelinated axons, and neurotrophic factor secretion from transplants reduces damage and rescues the spinal cord (Barnabe-Heider and Frisen, 2008). In chronic SCI, transplantation of NS/PCs expressing neurotrophin-3 induces motor function recovery (Kusano et al., 2010), supporting the significance of neurotrophic factors in restoration, even in the chronic stage. In the present study, however, GSI-treated hiPSC-NS/PCs did not show significant upregulation of neurotrophic factor secretion into culture medium compared with conventional hiPSC-NS/PCs. Therefore, the paracrine effect obtained from GSI-treated hiPSC-NS/PCs may not have been involved in the improvement of motor function recovery. With respect to the cell-autonomous function of graft-derived cells, GSI-treated hiPSC-NS/PCs differentiated into mature neurons, which integrated into the host neural circuitry, leading to inhibitory synaptic connection with host neurons. Therefore, our results suggested that GSI-treated hiPSC-NS/PCs mainly contribute to reconstruction of the neuronal circuitry and synaptic connection between the donor and host-derived neurons, rather than spinal cord preservation by secreted neurotrophic factors. It is possible that GSI transplants have enhanced response to neurotrophins, even without increased neurotrophin secretion.

GABAergic neurons present a neurotransmitter phenotype that plays an important role in the spinal cord by regulating primary afferent transmitter release and direct inhibitory postsynapses of motor neurons (Malcangio and Bowery, 1996; Schneider and Fyffe, 1992). A collapse of GABAergic activity after SCI causes severe spasticity and loss of motor coordination (Kim et al., 2010). Transplantation of GABAergic precursor cells into the spinal cord is known to inhibit neuropathic pain and promote functional improvement following SCI (Mukhida et al., 2007). The current study revealed that almost all the grafted cells were GABAergic and integrated with the host neural network to form inhibitory synapses. GSI-treated hiPSC-NS/PCs also resulted in a significantly longer stride length in the treadmill gait analyses and a significantly longer time on the rotarod during rotarod testing. These results indicated that the inhibitory synapse formation by transplanted cell-derived GABAergic neurons observed in the present study may contribute to the suppression of spasticity and improvement of motor coordination.

Ballermann and Fouad (2006) reported that locomotor recovery occurs in parallel with increased numbers of RtST fiber collaterals in rats subjected to lateral thoracic hemisection of the spinal cord. In the present study, our tracing analyses in the GSI-treated group revealed an increase of RtST fibers, and the number of the fibers was significantly correlated with the BMS score. Therefore, these results suggested that transplantation of GSI-treated hiPSC-NS/PCs may lead to an increased number of RtST fiber contacts with host internal neurons and recovery of motor function after transplantation in the chronic phase of SCI.

Our previous report showed that GSI-treated hiPSC-NS/PCs resulted in significantly greater functional recovery when cells were transplanted in subacute SCI (BMS score for the GSI (+) group = 4.9 ± 0.3 at 89 days post transplantation) (Okubo et al., 2016). However, compared with the

(F) Representative *in vivo* images of immunohistochemistry staining for phosphorylated p38 (Pp38) and Stem121 at the injury site. $Pp38^{+}$ cells contacted $Stem121^{+}$ transplanted cells. Scale bars, 20 μm.

(G) Percentages of Pp38-positive cells among the $Stem121^{+}$ transplanted cells at 84 days after transplantation (GSI (−) group, n = 10; GSI (+) group, n = 10 mice).

*p < 0.05, **p < 0.01 and N.S., non-significant according to the Wilcoxon rank-sum test. The average value in the GSI (−) group was normalized as 1 (n = 3 independent experiments). The data are presented as the means ± SEM.

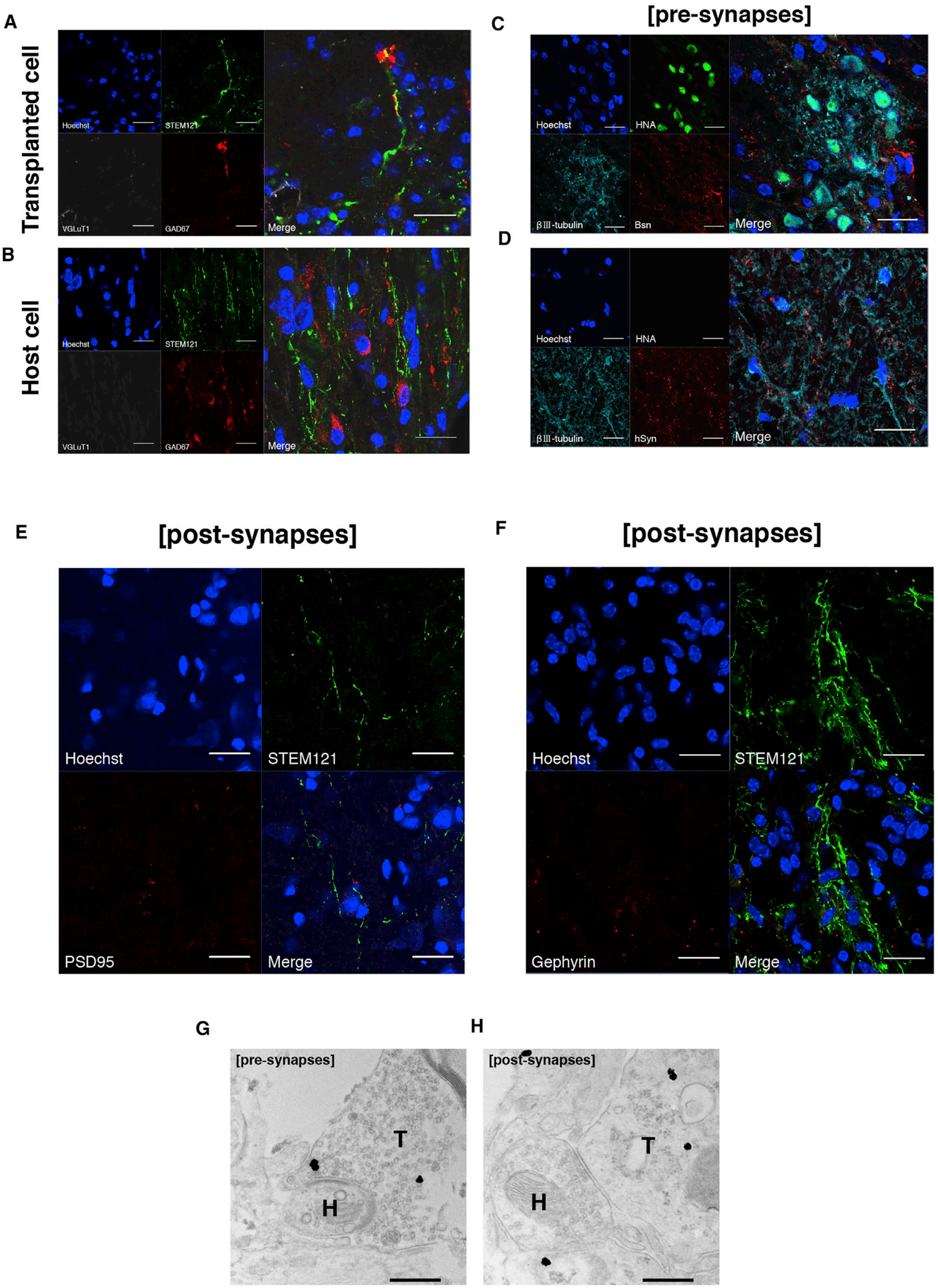

(legend on next page)

subacute phase, transplantation in the chronic stage resulted in slight improvement in the present study (BMS score for GSI (+) group: 3.1 ± 0.2 (201B7 transplantation) or 3.2 ± 1.1 (414C2 transplantation) at 84 days post transplantation). Because glial scar formation may be completed depending on the destructive damage to the chronically injured spinal cord, the effects of axonal regrowth obtained from cell transplantation alone may be restrictive. Environmental modulation of chronically injured spinal cords and modification of graft cells may be important for successful translation of stem cell-based therapies for the treatment of chronic SCI (Keirstead et al., 2005; Kumamaru et al., 2013; Nishimura et al., 2013). The solution to overcome this issue is likely combinatory therapy with rehabilitation. Tashiro et al. (2016) showed that treadmill training combined with NS/PC transplantation in the chronic stage promotes significant recovery of motor function, which they attributed to neuronal differentiation and maturation of the CPG. However, this combined treatment may not sufficiently repair the lesion epicenter, depending on the severity of glial scar formation. Tashiro et al. (2016) also reported that there were no significant differences in residual spinal volume, $5HT^+$ serotonergic fibers, or synaptic generation before and after therapy. Because GSI treatment leads to axonal regrowth and remyelination even at the lesion epicenter, combination therapy involving GSI-treated cell transplantation and rehabilitative treadmill training may be an ideal treatment for the chronic phase of SCI to achieve improved results by compensating for the weakness of each therapy.

Recently, a first-in-humans phase I clinical trial was performed in patients with chronic SCI using human spinal cord-derived neural stem cells (NSI-566) authorized by the FDA for clinical testing, primarily to demonstrate safety (Curtis et al., 2018), which represents a step toward establishing stem cell therapy for chronic SCI. As a next step in clinical trials for patients with chronic SCI, our primary goals are to restore the host neuronal circuitry at the injured spinal cord through newly formed synapses between donor and host-derived neurons and to achieve regrowth of motor and sensory axons generated from grafted cells. It will be important that the GSI-treated hiPSC-NS/PCs successfully reconstruct the neuronal circuitry and synaptic connections between the grafted neurons and host neurons after transplantation not only in the subacute phase (Okubo et al., 2016), but also in the chronic phase of SCI (as in the present study). Thus, treatment of cells with GSI prior to transplantation is a simple and clinically applicable method that may be useful for enhancing the safety and efficacy of transplantation therapy in SCI.

In conclusion, treatment of hiPSC-NS/PCs with GSI only 1 day before transplantation leads to significantly greater axonal regrowth, remyelination by host-derived glial cells, and integration with the host neural circuitry through inhibitory synapse formation. These findings contribute to the recovery of motor function without deterioration even after transplantation in the chronic phase of SCI.

EXPERIMENTAL PROCEDURES

Human iPSCs

All the hiPSC experiments were performed using NS/PCs derived from the following two cell lines: normal clone 201B7 (Miura et al., 2009), generated following retroviral transfection of four factors (Oct3/4, Sox2, Klf4, and C-Myc); and clone 414C2 (Okita et al., 2011), reprogrammed with episomal plasmid vectors containing six factors (Oct3/4, Sox2, Klf4, L-Myc, LIN28, and p53shRNA).

Mice

For the *in vivo* transplantation experiment, adult female NOD-SCID (NOD/ShiJic-*scid*Jcl; mice, 20–22 g; Clea, Tokyo, Japan) were housed in sterile housing with four to five animals per cage.

Figure 5. Transplanted GSI-Treated hiPSC-NS/PCs Contribute to Inhibitory Synapse Formation between Transplanted Cell-Derived Mature Neurons and Host Mouse Neurons

(A and B) GSI-treated hiPSC-NS/PCs ($STEM121^+$ transplanted cells) differentiated into $GAD67^+$ (GABAergic) neurons. (A) $STEM121^+$ transplanted cells contacted (B) $STEM121^-/GAD67^+$ host mouse cells.

(C) Representative images of immunohistochemistry staining for HNA, βIII-tubulin, and Bassoon (Bsn) (mouse presynaptic marker). βIII-$tubulin^+/HNA^+$ transplanted cell-derived neurons contacted Bsn^+ cells.

(D) Representative images of immunohistochemistry staining for HNA, βIII-tubulin, and hSyn (human-specific presynaptic marker). $hSyn^+$ boutons apposed β III-$tubulin^+/HNA^-$ host mouse neurons.

(E) Representative images of immunohistochemistry staining for STEM121 and postsynaptic density protein 95 (PSD95) (a marker of postexcitatory synaptic boutons). $PSD95^+$ boutons were rare.

(F) Representative images of immunohistochemistry staining for STEM121 and Gephryin (a marker of postinhibitory synaptic boutons). $Gephryin^+$ boutons apposed $STEM121^+$ cells.

(G and H) Representative immunoelectron microscopy images show synapse formation between transplant-derived $STEM121^+$ (black dots) human neurons and host mouse neurons. The pre- (G) and postsynaptic (H) structures indicated transmission from a host neuron to a transplant-derived neuron. T, transplant-derived neuron; H, host neuron.

Scale bars, 20 μm in (A–F) and 500 nm in (G and H).

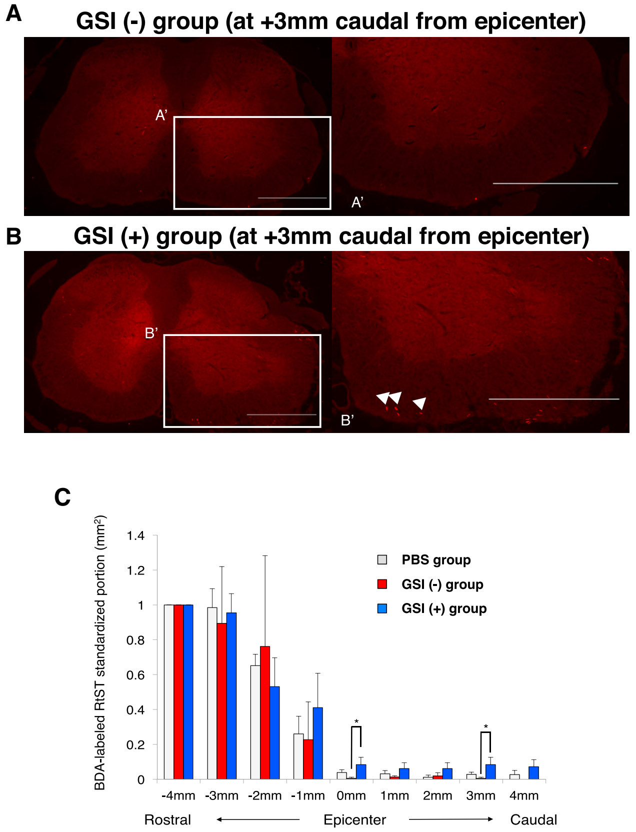

Figure 6. Transplantation of GSI-Treated hiPSC-NS/PCs Increases the RtST

(A and B) Representative images of immunohistochemistry staining for BDA-labeled RtST fibers in axial spinal cord sections at a site +3 mm caudal to the epicenter: (A) GSI (−) group, (B) GSI (+) group, (A′) boxed area in (A), (B′) boxed area in (B). White arrow, RtST fiber. Scale bars, 500 μm.

(C) Quantification of the BDA-positive area. The GSI (+) group displayed extension of RtST-positive fibers even at a site caudal to the epicenter. $^*p < 0.05$ according to one-way ANOVA with the Tukey-Kramer test. PBS group, n = 4; GSI (−) group, n = 6; GSI (+) group, n = 4 mice.

Eight-week-old NOD/SCID mice were subjected to *in vivo* SCI and cell transplantation experiments. All experiments were performed in accordance with the Guidelines for the Care and Use of Laboratory Animals of Keio University (Assurance no. 13,020) and the NIH Guide for the Care and Use of Laboratory Animals.

Cell Culture, NS/PCs Derived from hiPSC Formation

hiPSCs were cultured for 12 days in adhesion cultures with mouse embryonic fibroblasts and then formed embryo bodies upon floating culture for 30 days. Aggregate cells were differentiated into NS/PCs derived from hiPSC-NS/PC formation using various factors during each day of the incubation period. Detailed methods are previously described (Okada et al., 2008) with slight modifications.

Treatment of hiPSC-NS/PCs with GSI

Treatment of hiPSC-NS/PCs with GSI was performed as described previously (Okubo et al., 2016), and detailed methods are provided in Supplemental Experimental Procedures.

SCI Animal Model and Cell Transplantation

Adult female NOD-SCID mice were anesthetized through intraperitoneal (i.p.) injection of ketamine (100 mg/kg) and xylazine (10 mg/kg). After laminectomy at the level of the tenth thoracic spinal vertebra, the dorsal surface of the dura mater was exposed, and contusive SCI was performed using an IH impactor (a force defined impact [60 kdyn] with a stainless steel-tipped impactor; Precision Systems and Instrumentation, Lexington, KY, USA) as described previously (Scheff et al., 2003). Donor cells were prepared *in vitro* from 201B7 to 414C2 hiPSC-NS/PCs, which were cultured with GSI for 1 day in the "GSI (+) group" and without GSI in "GSI (−) group" before cell transplantation. At 42 days after SCI, hiPSC-NS/PCs treated with or without GSI (5×10^5 cells/2 μL) were transplanted into the lesion epicenter of each mouse (GSI (−) group and GSI (+) group, n = 10 each) with a glass micropipette at a rate of 1 μL/min using a Hamilton syringe (25 μL) and a stereotaxic micro injector (KDS 310; Muromachi Kikai, Tokyo, Japan). An equal volume of PBS was injected for the "PBS group" mice.

Lentiviral Infection

In brief, two hiPSC-NS/PC lines were dissociated and infected with a lentivirus expressing ffLuc, a fusion protein of firefly luciferase and Venus fluorescent protein under the control of the EF promoter to enable the detection of transplanted cells through their bioluminescent ffLuc signals in live SCI mice and in fixed spinal cord sections.

BLI Imaging

A Xenogen-IVIS spectrum cooled, charge-coupled device optical macroscopic imaging system (Caliper Life-Sciences, Hopkinton, MA, USA) was used for BLI to evaluate the survival of transplanted hiPSC-NS/PCs as described previously (Itakura et al., 2015; Okada et al., 2005; Takahashi et al., 2011). The integration time was fixed at 5 min for each image. Imaging was performed for 5 min *in vivo* after i.p. injection of D-luciferin (0.3 mg/g body weight, Promega, Madison, WI, USA) with the field-of-view set at 13.2 cm, as the photon count was most stable during this period. The intensity peaked between 10 and 30 min. All images were measured with Living Image software, and the optical signal intensity was expressed as the photon count in units of photons/s/cm^2/str. Each result was displayed as a pseudo-colored photon count image superimposed on a gray-scale anatomic image. A region of interest was defined in the cell-implanted area, and all values in the same region of interest were elucidated to quantify the light measured. The survival of cells transplanted in the mouse spinal cord was measured by BLI using the IVIS system every week until 84 days after transplantation.

Histological Analyses

Histological analyses were performed at 84 days after transplantation. Spinal cord sections were histologically evaluated by staining with H&E and LFB and through immunohistochemistry (IHC).

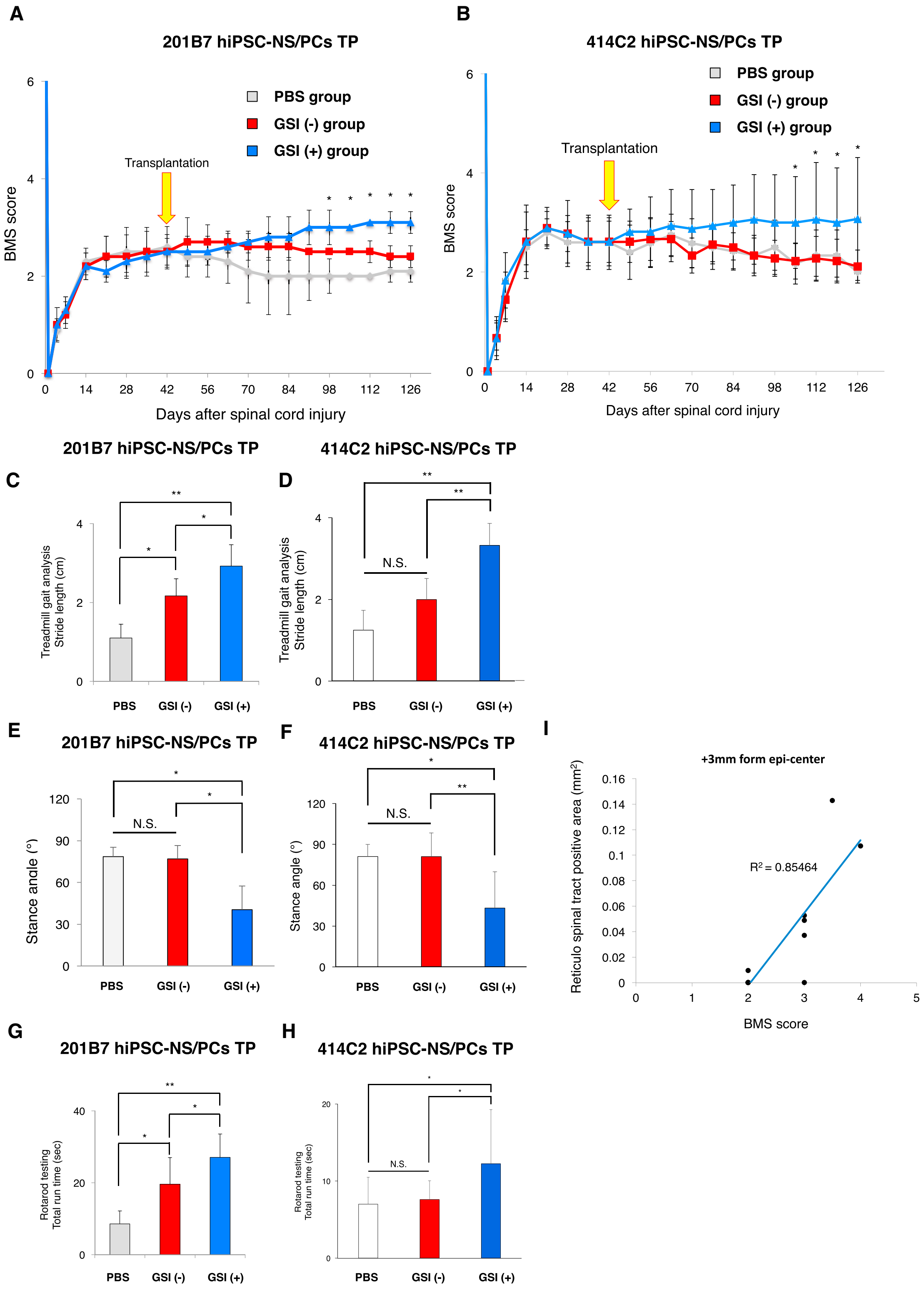

Figure 7. Long-Term Motor Function Analyses after Transplantation of GSI-Treated hiPSC-NS/PCs

(A and B) Comparison of BMS scores among the PBS, GSI (−), and GSI (+) groups. Motor function in the hind limbs was assessed weekly for up to 84 days after transplantation in the chronic phase of SCI using the BMS score. (A) 201B7 cell line; PBS group, n = 10; GSI (−) group, n = 10; GSI (+) group, n = 10 mice; and (B) 414C2 cell line; PBS group, n = 10; GSI (−) group, n = 10; GSI (+) group, n = 10 mice.

(legend continued on next page)

Detailed protocols are provided in Supplemental Experimental Procedures.

Immunoelectron Microscopy Analyses

Frozen sections of mouse axial spinal cords with transplanted human cells were incubated with 5% block ace (DS Pharma Biomedical) with 0.01% Saponin in 0.1 M PBS for 1 hr. The sections were then stained with a primary mouse anti-human cytoplasm antibody (STEM121; mouse immunoglobulin G1 (IgG1), 1:500; Takara Bio, Kusatsu, Japan, Y40410) for 72 hr at 4°C, followed by incubation with a nanogold-conjugated goat anti-mouse secondary antibody (1:100; Thermo Fisher Scientific, Waltham, MA, USA, N-24915) for 24 hr at 4°C. After 2.5% glutaraldehyde fixation, nanogold signals were enhanced with R-Gent SE-EM Silver Enhancement Reagents (Aurion) for 30 min. Thereafter, the sections were fixed with 1.0% OsO_4 for 90 min at 25°C, dehydrated through a graded series of ethanol, acetone, and QY1 and embedded in Epon. Polymerization was performed for 72 hr at 60°C in an EM oven (DSK, Osaka, Japan). Ultrathin sections (70 nm) were prepared with an ultramicrotome (Leica UC7) and electron stained with uranyl acetate and lead citrate. The ultrathin sections were finally observed under a transmission EM (JEOL model 1,400 plus, Tokyo, Japan).

Cytokine Array

To obtain conditioned medium, GSI-treated hiPSC-NS/PCs and conventional NS/PCs were cultured separately in growth medium. At 48 hr before collection, the cells were washed once with proliferation medium consisting of serum-free medium, and the medium was replaced with fresh growth medium. For background subtraction, the same amount of growth medium without cells was also subjected to incubation. The culture media were collected after 48 hr, centrifuged to remove cell debris, and stored at −80°C. The number of viable cells in each culture was calculated for normalization. Cytokine profiles were determined using human quantitative custom antibody arrays (RayBiotech, Norcross, GA, USA) to detect nine cytokines (BDNF, b-NGF, HGF, CTNF, GDNF, NT-3, NT-4, PDGF-AA, and VEGF), which were measured and processed according to the manufacturer's recommendation. The antibody array was a glass-chip-based multiplexed sandwich enzyme-linked immunosorbent assay system. A standard glass slide was spotted with 16 wells of identical biomarker antibody arrays. Each antibody, together with the positive and negative controls, was arrayed in quadruplicate. The samples and standards were added to the wells of the chip array, followed by incubation overnight at 4°C, then five washing steps and the addition of a biotinylated antibody and Cy3 equivalent dye labeled-streptavidin to the wells. The signals were scanned and extracted with InnoScan 710 and Mapix software (Innopsys, Carbonne, France). After background subtraction, concentrations were calculated against a standard curve for each biomarker generated from the positive and negative controls using Quantibody Analyzer software (RayBiotech). Cytokine release into culture supernatants was normalized according to the number of viable cells in each culture.

Neuronal Differentiation and Quantification of Neurite Outgrowth

SB203580, a p38 MAPK inhibitor, was dissolved in DMSO at a final concentration of 1 mM. hiPSC-NS/PCs were cultured *in vitro* with GSI alone, a p38 MAPK inhibitor alone or with both for 1 day. For neuronal differentiation, cells were plated onto poly-L-ornithine/fibronectin-coated 48-well chamber slides (Costar 3548; Corning, NY, USA) at a density of 1×10^5 cells/mL and cultured in medium without growth factors at 37°C in 5% CO_2 and 95% air for 14 days. At 7 or 14 days after neuronal differentiation, cells were fixed with 4% paraformaldehyde (PFA) in 0.1 M PBS, and the length of neurite extension from each cell was measured (n = 10 per group).

Western Blotting

SDS-PAGE and western blotting were performed following standard protocols according to the manufacturer's instructions. The primary antibodies employed in these assays were phospho-p38 MAPK (rabbit IgG, 1:500; Cell Signaling Technology, Danvers, MA, USA, 8690L) and β-actin (mouse IgG, 1:500; Sigma-Aldrich, St. Louis, MO, USA, A1978-100UL). The secondary antibody was horseradish peroxidase-conjugated goat anti-rabbit IgG (1:5,000, Thermo Fisher Scientific, 65–6120). Immunoreactive bands were detected using an enhanced chemiluminescence kit (Thermo Fisher Scientific USA).

Anterograde and *trans*-Synaptic Labeling of the RtST

Mice were anaesthetized and placed in a stereotaxic frame. Pressure injection (50 nL) of BDA (10,000 MW; Molecular Probes; 10% in DW) was performed at the reticular formation (depth 3.4–4.85 mm; lateral 0–1.25 mm; and rostral 5.63–7.19 mm) as described previously (Liang et al., 2016). Two weeks after BDA

(C–F) Comparison of stride lengths and stance angles among the PBS, GSI (−), and GSI (+) groups. Treadmill gait analyses were performed at 84 days after transplantation in the chronic phase SCI using the DigiGait system. Histograms show the results regarding stride length (C and D) and stance angle (E and F) (201B7 cell line; PBS group, n = 10; GSI (−) group, n = 10; GSI (+) group, n = 10 mice, 414C2 cell line; PBS group, n = 10; GSI (−) group, n = 10; GSI (+) group, n = 10 mice).

(G and H) Comparison of the results of the rotarod test among the PBS, GSI (−), and GSI (+) groups. The rotarod test was performed at 84 days after transplantation in the chronic phase of SCI. Histograms show the total run time (G) 201B7 cell line; PBS group, n = 10; GSI (−) group, n = 10; GSI (+) group, n = 10 mice (H) 414C2 cell line; PBS group, n = 10; GSI (−) group, n = 10; GSI (+) group, n = 10 mice.

(I) Scatterplots comparing the BMS score and the BDA-labeled RtST standardized portion at a site 3 mm caudal to the epicenter (n = 14 mice). A significant correlation was revealed at the epicenter and +3 mm caudal site. The correlation coefficient was highest at +3 mm caudal to the epicenter.

*$p < 0.05$, **$p < 0.01$, or not significant (N.S.) according to two-way repeated-measures ANOVA with the Tukey-Kramer test (A and B) or one-way ANOVA with the Tukey-Kramer test (C–H). Data are presented as means ± SEM.

injection, the animals were anesthetized and transcardially euthanized with 0.1 M PBS containing 4% PFA. Their spinal cords were then removed, postfixed overnight in 4% PFA, soaked overnight in 10% sucrose, followed by 30% sucrose for several days, embedded in OCT compound, frozen, and sectioned along the axial plane at a 14 μm thickness on a cryostat. Images were obtained using a fluorescence microscope (BZ-X710).

Behavioral Analyses

Two investigators blinded to the identities of the experimental mice performed this assessment. Motor coordination was evaluated using a rotating rod apparatus (rotarod, Muromachikikai), which consisted of a plastic rod (3 cm diameter and 8 cm length) with a gridded surface flanked by two large discs (40 cm diameter). Each mouse was placed on the rod while it was rotated at 20 rpm in 2-min sessions at 84 days after transplantation. Five trials were conducted, and the maximum number of seconds the mouse stayed on the rod was recorded. Gait analyses were performed using the DigiGait system (Mouse Specifics, Quincy, MA, USA; n = 5 per group). Each mouse exhibited weight-supported hindlimb stepping at 84 days after transplantation. Stride length and stance angle were determined on a treadmill set to a speed of 7 cm/s.

Quantification and Statistical Analyses

All data are reported as the means ± SEM. The Wilcoxon rank-sum test was used to evaluate the differences between groups with respect to the BLI analyses, H&E staining, IHC, western blotting and cytokine assays. One-way ANOVA followed by the Tukey-Kramer test for multiple comparisons was used to evaluate the differences in the transverse area of the spinal cord for H&E or LFB staining; IHC results for NF-H and 5-HT; quantification of neurite outgrowth; BDA-labeled RtST standardized portion; rotarod results; and DigiGait results. Two-way repeated measures ANOVA tests followed by the Tukey-Kramer test was used for BMS analyses. p values < 0.05 or <0.01 were considered to indicate statistical significance.

SUPPLEMENTAL INFORMATION

Supplemental Information includes Supplemental Experimental Procedures and four figures can be found with this article online at https://doi.org/10.1016/j.stemcr.2018.10.022.

AUTHOR CONTRIBUTIONS

T.O. designed the project, performed most of the experiments, interpreted the data, and wrote the manuscript, with technical assistance from N.N. and J.K. O.T., M.S., S.S., Y.K., and M.M. provided experimental support and ideas for the project. M.N. and H.O. designed the studies, supervised the overall project, and prepared the final manuscript.

ACKNOWLEDGMENTS

We appreciate the assistance of Drs. K. Kojima, S. Ito, Y Tanimoto, and Y Hoshino, who are all members of the Spinal Cord Research Team in the Department of Orthopedic Surgery, Keio University School of Medicine, Tokyo, Japan. We thank Prof. Shinya Yamanaka for the human iPSC clones (201B7 and 414C2). We also thank Ms. T. Harada, Ms. K. Yasutake, and Ms. M. Akizawa for their assistance with the experiments and animal care. This work was supported by the Research Center Network for Realization of Regenerative Medicine of the Japan Agency for Medical Research and Development (AMED) (grant no. 15bm0204001h0003 to H.O. and M.N.), and by a medical research grant related to traffic accidents from the General Insurance Association of Japan. H.O. is a compensated scientific consultant of San Bio, and K Pharma, and M.N. is a compensated scientific consultant of K Pharma.

Received: October 19, 2018
Revised: October 29, 2018
Accepted: October 29, 2018
Published: November 29, 2018

REFERENCES

Ballermann, M., and Fouad, K. (2006). Spontaneous locomotor recovery in spinal cord injured rats is accompanied by anatomical plasticity of reticulospinal fibers. Eur. J. Neurosci. *23*, 1988–1996.

Barnabe-Heider, F., and Frisen, J. (2008). Stem cells for spinal cord repair. Cell Stem Cell *3*, 16–24.

Basso, D.M., Fisher, L.C., Anderson, A.J., Jakeman, L.B., McTigue, D.M., and Popovich, P.G. (2006). Basso Mouse Scale for locomotion detects differences in recovery after spinal cord injury in five common mouse strains. J. Neurotrauma *23*, 635–659.

Cummings, B.J., Uchida, N., Tamaki, S.J., Salazar, D.L., Hooshmand, M., Summers, R., Gage, F.H., and Anderson, A.J. (2005). Human neural stem cells differentiate and promote locomotor recovery in spinal cord-injured mice. Proc. Natl. Acad. Sci. U S A *102*, 14069–14074.

Curtis, E., Martin, J.R., Gabel, B., Sidhu, N., Rzesiewicz, T.K., Mandeville, R., Van Gorp, S., Leerink, M., Tadokoro, T., Marsala, S., et al. (2018). A first-in-human, phase I study of neural stem cell transplantation for chronic spinal cord injury. Cell Stem Cell *22*, 941–950.e6.

Garcia-Alias, G., Truong, K., Shah, P.K., Roy, R.R., and Edgerton, V.R. (2015). Plasticity of subcortical pathways promote recovery of skilled hand function in rats after corticospinal and rubrospinal tract injuries. Exp. Neurol. *266*, 112–119.

Ghosh, M., and Pearse, D.D. (2014). The role of the serotonergic system in locomotor recovery after spinal cord injury. Front. Neural Circuits *8*, 151.

Hara-Miyauchi, C., Tsuji, O., Hanyu, A., Okada, S., Yasuda, A., Fukano, T., Akazawa, C., Nakamura, M., Imamura, T., Matsuzaki, Y., et al. (2012). Bioluminescent system for dynamic imaging of cell and animal behavior. Biochem. Biophys. Res. Commun. *419*, 188–193.

Itakura, G., Kobayashi, Y., Nishimura, S., Iwai, H., Takano, M., Iwanami, A., Toyama, Y., Okano, H., and Nakamura, M. (2015). Control of the survival and growth of human glioblastoma grafted into the spinal cord of mice by taking advantage of immunorejection. Cell Transplant *24*, 1299–1311.

Ito, M., Natsume, A., Takeuchi, H., Shimato, S., Ohno, M., Wakabayashi, T., and Yoshida, J. (2009). Type I Interferon inhibits astrocytic gliosis and promotes functional recovery after spinal cord

injury by deactivation of the MEK/ERK pathway. J. Neurotrauma *26*, 41–53.

Karimi-Abdolrezaee, S., Eftekharpour, E., Wang, J., Schut, D., and Fehlings, M.G. (2010). Synergistic effects of transplanted adult neural stem/progenitor cells, chondroitinase, and growth factors promote functional repair and plasticity of the chronically injured spinal cord. J. Neurosci. *30*, 1657–1676.

Kawabata, S., Takano, M., Numasawa-Kuroiwa, Y., Itakura, G., Kobayashi, Y., Nishiyama, Y., Sugai, K., Nishimura, S., Iwai, H., Isoda, M., et al. (2016). Grafted human iPS cell-derived oligodendrocyte precursor cells contribute to robust remyelination of demyelinated axons after spinal cord injury. Stem Cell Reports *6*, 1–8.

Keirstead, H.S., Nistor, G., Bernal, G., Totoiu, M., Cloutier, F., Sharp, K., and Steward, O. (2005). Human embryonic stem cell-derived oligodendrocyte progenitor cell transplants remyelinate and restore locomotion after spinal cord injury. J. Neurosci. *25*, 4694–4705.

Kim, D.S., Jung, S.J., Nam, T.S., Jeon, Y.H., Lee, D.R., Lee, J.S., Leem, J.W., and Kim, D.W. (2010). Transplantation of GABAergic neurons from ESCs attenuates tactile hypersensitivity following spinal cord injury. Stem Cells *28*, 2099–2108.

Kondoh, K., Sunadome, K., and Nishida, E. (2007). Notch signaling suppresses p38 MAPK activity via induction of MKP-1 in myogenesis. J. Biol. Chem. *282*, 3058–3065.

Kopan, R., and Ilagan, M.X. (2004). Gamma-secretase: proteasome of the membrane? Nat. Rev. Mol. Cell Biol. *5*, 499–504.

Kumamaru, H., Saiwai, H., Kubota, K., Kobayakawa, K., Yokota, K., Ohkawa, Y., Shiba, K., Iwamoto, Y., and Okada, S. (2013). Therapeutic activities of engrafted neural stem/precursor cells are not dormant in the chronically injured spinal cord. Stem Cells *31*, 1535–1547.

Kusano, K., Enomoto, M., Hirai, T., Tsoulfas, P., Sotome, S., Shinomiya, K., and Okawa, A. (2010). Transplanted neural progenitor cells expressing mutant NT3 promote myelination and partial hindlimb recovery in the chronic phase after spinal cord injury. Biochem. Biophys. Res. Commun. *393*, 812–817.

Li, S., Kim, J.E., Budel, S., Hampton, T.G., and Strittmatter, S.M. (2005). Transgenic inhibition of Nogo-66 receptor function allows axonal sprouting and improved locomotion after spinal injury. Mol. Cell. Neurosci. *29*, 26–39.

Liang, H., Watson, C., and Paxinos, G. (2016). Terminations of reticulospinal fibers originating from the gigantocellular reticular formation in the mouse spinal cord. Brain Struct. Funct. *221*, 1623–1633.

Malcangio, M., and Bowery, N.G. (1996). GABA and its receptors in the spinal cord. Trends Pharmacol. Sci. *17*, 457–462.

May, Z., Fenrich, K.K., Dahlby, J., Batty, N.J., Torres-Espin, A., and Fouad, K. (2017). Following spinal cord injury transected reticulospinal tract axons develop new collateral inputs to spinal interneurons in parallel with locomotor recovery. Neural Plast. *2017*, 1932875.

Miura, K., Okada, Y., Aoi, T., Okada, A., Takahashi, K., Okita, K., Nakagawa, M., Koyanagi, M., Tanabe, K., Ohnuki, M., et al. (2009). Variation in the safety of induced pluripotent stem cell lines. Nat. Biotechnol. *27*, 743–745.

Mothe, A.J., and Tator, C.H. (2013). Review of transplantation of neural stem/progenitor cells for spinal cord injury. Int. J. Dev. Neurosci. *31*, 701–713.

Mukhida, K., Mendez, I., McLeod, M., Kobayashi, N., Haughn, C., Milne, B., Baghbaderani, B., Sen, A., Behie, L.A., and Hong, M. (2007). Spinal GABAergic transplants attenuate mechanical allodynia in a rat model of neuropathic pain. Stem Cells *25*, 2874–2885.

Nishimura, S., Yasuda, A., Iwai, H., Takano, M., Kobayashi, Y., Nori, S., Tsuji, O., Fujiyoshi, K., Ebise, H., Toyama, Y., et al. (2013). Time-dependent changes in the microenvironment of injured spinal cord affects the therapeutic potential of neural stem cell transplantation for spinal cord injury. Mol. Brain *6*, 3.

Nix, P., Hisamoto, N., Matsumoto, K., and Bastiani, M. (2011). Axon regeneration requires coordinate activation of p38 and JNK MAPK pathways. Proc. Natl. Acad. Sci. U S A *108*, 10738–10743.

Nori, S., Okada, Y., Nishimura, S., Sasaki, T., Itakura, G., Kobayashi, Y., Renault-Mihara, F., Shimizu, A., Koya, I., Yoshida, R., et al. (2015). Long-term safety issues of iPSC-based cell therapy in a spinal cord injury model: oncogenic transformation with epithelial-mesenchymal transition. Stem Cell Reports *4*, 360–373.

Nori, S., Okada, Y., Yasuda, A., Tsuji, O., Takahashi, Y., Kobayashi, Y., Fujiyoshi, K., Koike, M., Uchiyama, Y., Ikeda, E., et al. (2011). Grafted human-induced pluripotent stem-cell-derived neurospheres promote motor functional recovery after spinal cord injury in mice. Proc. Natl. Acad. Sci. U S A *108*, 16825–16830.

Okada, S., Nakamura, M., Mikami, Y., Shimazaki, T., Mihara, M., Ohsugi, Y., Iwamoto, Y., Yoshizaki, K., Kishimoto, T., Toyama, Y., et al. (2004). Blockade of interleukin-6 receptor suppresses reactive astrogliosis and ameliorates functional recovery in experimental spinal cord injury. J. Neurosci. Res. *76*, 265–276.

Okada, S., Ishii, K., Yamane, J., Iwanami, A., Ikegami, T., Katoh, H., Iwamoto, Y., Nakamura, M., Miyoshi, H., Okano, H.J., et al. (2005). In vivo imaging of engrafted neural stem cells: its application in evaluating the optimal timing of transplantation for spinal cord injury. FASEB J. *19*, 1839–1841.

Okada, Y., Matsumoto, A., Shimazaki, T., Enoki, R., Koizumi, A., Ishii, S., Itoyama, Y., Sobue, G., and Okano, H. (2008). Spatiotemporal recapitulation of central nervous system development by murine embryonic stem cell-derived neural stem/progenitor cells. Stem Cells *26*, 3086–3098.

Okano, H., Nakamura, M., Yoshida, K., Okada, Y., Tsuji, O., Nori, S., Ikeda, E., Yamanaka, S., and Miura, K. (2013). Steps toward safe cell therapy using induced pluripotent stem cells. Circ. Res. *112*, 523–533.

Okita, K., Matsumura, Y., Sato, Y., Okada, A., Morizane, A., Okamoto, S., Hong, H., Nakagawa, M., Tanabe, K., Tezuka, K., et al. (2011). A more efficient method to generate integration-free human iPS cells. Nat. Methods *8*, 409–412.

Okubo, T., Iwanami, A., Kohyama, J., Itakura, G., Kawabata, S., Nishiyama, Y., Sugai, K., Ozaki, M., Iida, T., Matsubayashi, K., et al. (2016). Pretreatment with a gamma-secretase inhibitor prevents tumor-like overgrowth in human iPSC-derived transplants for spinal cord injury. Stem Cell Reports *7*, 649–663.

Salewski, R.P., Mitchell, R.A., Li, L., Shen, C., Milekovskaia, M., Nagy, A., and Fehlings, M.G. (2015). Transplantation of induced

pluripotent stem cell-derived neural stem cells mediate functional recovery following thoracic spinal cord injury through remyelination of axons. Stem Cells Transl. Med. *4*, 743–754.

Scheff, S.W., Rabchevsky, A.G., Fugaccia, I., Main, J.A., and Lumpp, J.E. (2003). Experimental modeling of spinal cord injury: characterization of a force-defined injury device. J. Neurotrauma *20*, 179–193.

Schneider, S.P., and Fyffe, R.E.W. (1992). Involvement of GABA and glycine in recurrent inhibition of spinal motoneurons. J. Neurophysiol. *68*, 397–406.

Springer, J.E., Rao, R.R., Lim, H.R., Cho, S.I., Moon, G.J., Lee, S.Y., Park, E.J., Noh, J.S., and Gwag, B.J. (2010). The functional and neuroprotective actions of Neu2000, a dual-acting pharmacological agent, in the treatment of acute spinal cord injury. J. Neurotrauma *27*, 139–149.

Suzuki, H., Ahuja, C.S., Salewski, R.P., Li, L., Satkunendrarajah, K., Nagoshi, N., Shibata, S., and Fehlings, M.G. (2017). Neural stem cell mediated recovery is enhanced by chondroitinase ABC pretreatment in chronic cervical spinal cord injury. PLoS One *12*, e0182339.

Takahashi, Y., Tsuji, O., Kumagai, G., Hara, C.M., Okano, H.J., Miyawaki, A., Toyama, Y., Okano, H., and Nakamura, M. (2011). Comparative study of methods for administering neural stem/progenitor cells to treat spinal cord injury in mice. Cell Transplant *20*, 727–739.

Tashiro, S., Nishimura, S., Iwai, H., Sugai, K., Zhang, L., Shinozaki, M., Iwanami, A., Toyama, Y., Liu, M., Okano, H., et al. (2016). Functional recovery from neural stem/progenitor cell transplantation combined with treadmill training in mice with chronic spinal cord injury. Sci. Rep. *6*, 30898.

Stem Cell Reports, Volume *11*

Supplemental Information

Treatment with a Gamma-Secretase Inhibitor Promotes Functional Recovery in Human iPSC- Derived Transplants for Chronic Spinal Cord Injury

Toshiki Okubo, Narihito Nagoshi, Jun Kohyama, Osahiko Tsuji, Munehisa Shinozaki, Shinsuke Shibata, Yoshitaka Kase, Morio Matsumoto, Masaya Nakamura, and Hideyuki Okano

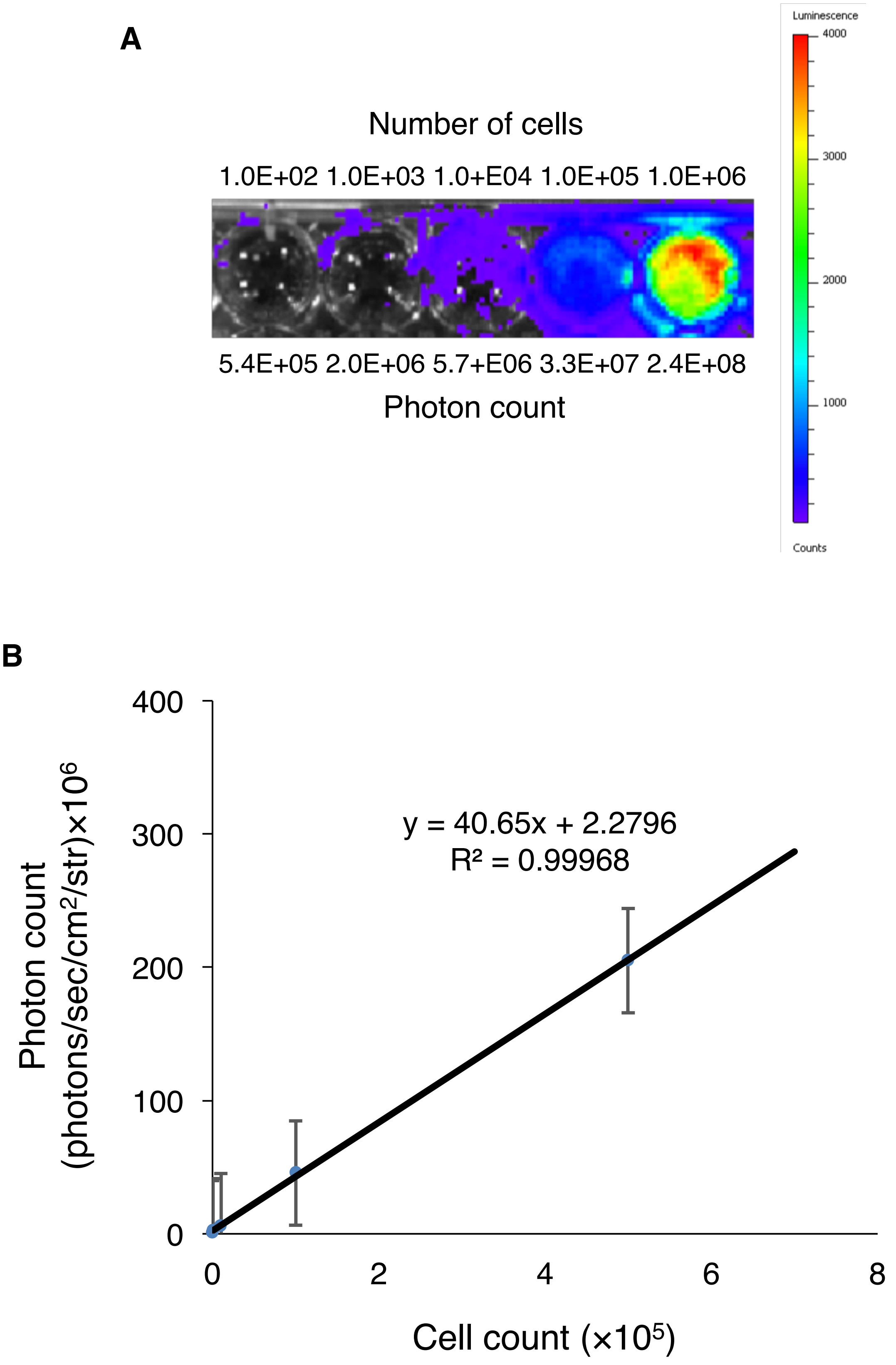
A
Number of cells
1.0E+02 1.0E+03 1.0+E04 1.0E+05 1.0E+06
5.4E+05 2.0E+06 5.7+E06 3.3E+07 2.4E+08
Photon count
Luminescence
4000
3000
2000
1000
Counts
B
400
300
200
100
0
y = 40.65x + 2.2796
R² = 0.99968
Photon count
(photons/sec/cm²/str)×10⁶
0
2
4
6
8
Cell count (×10⁵)

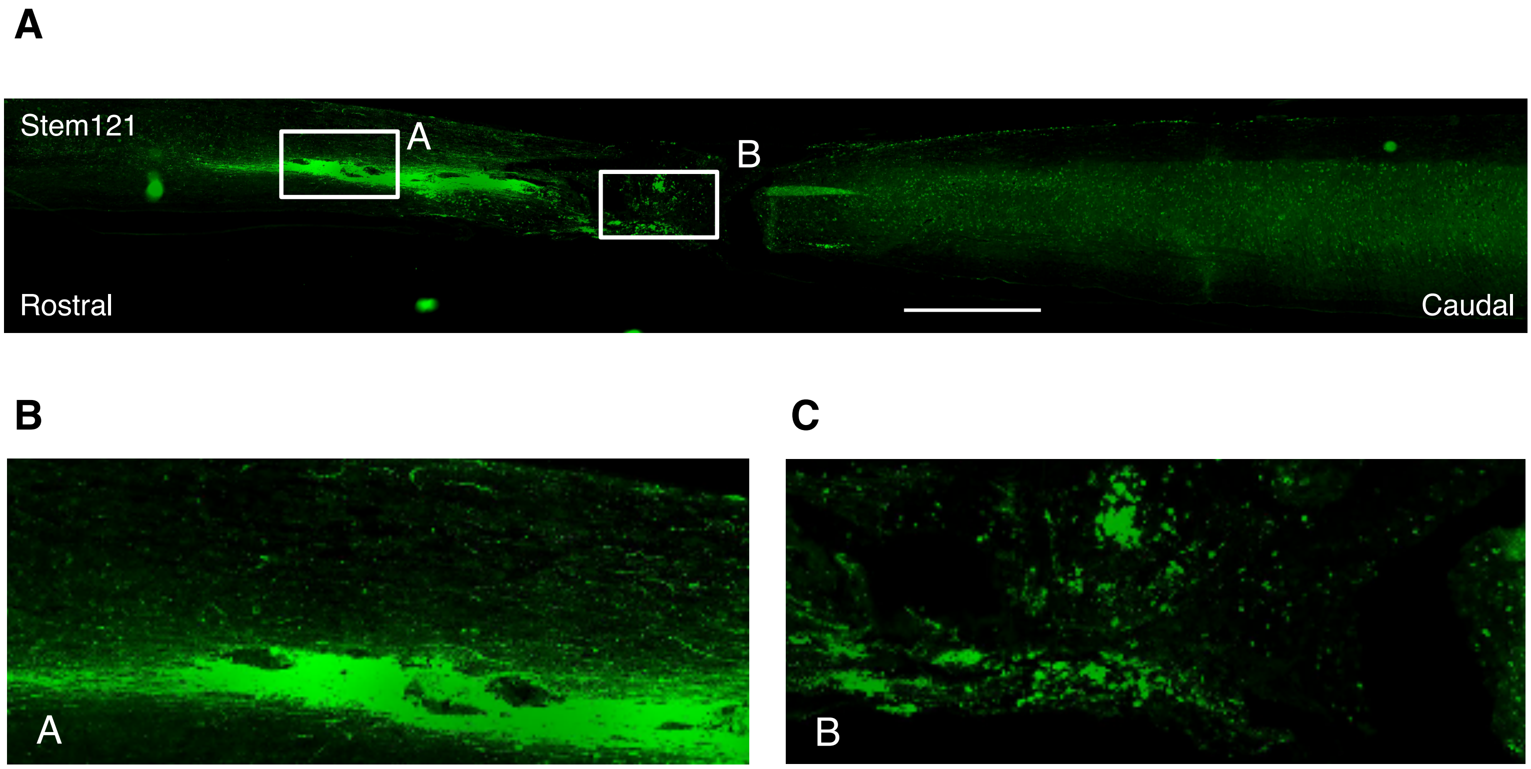
A
Stem121
A
B
Rostral
Caudal
B
C
A
B

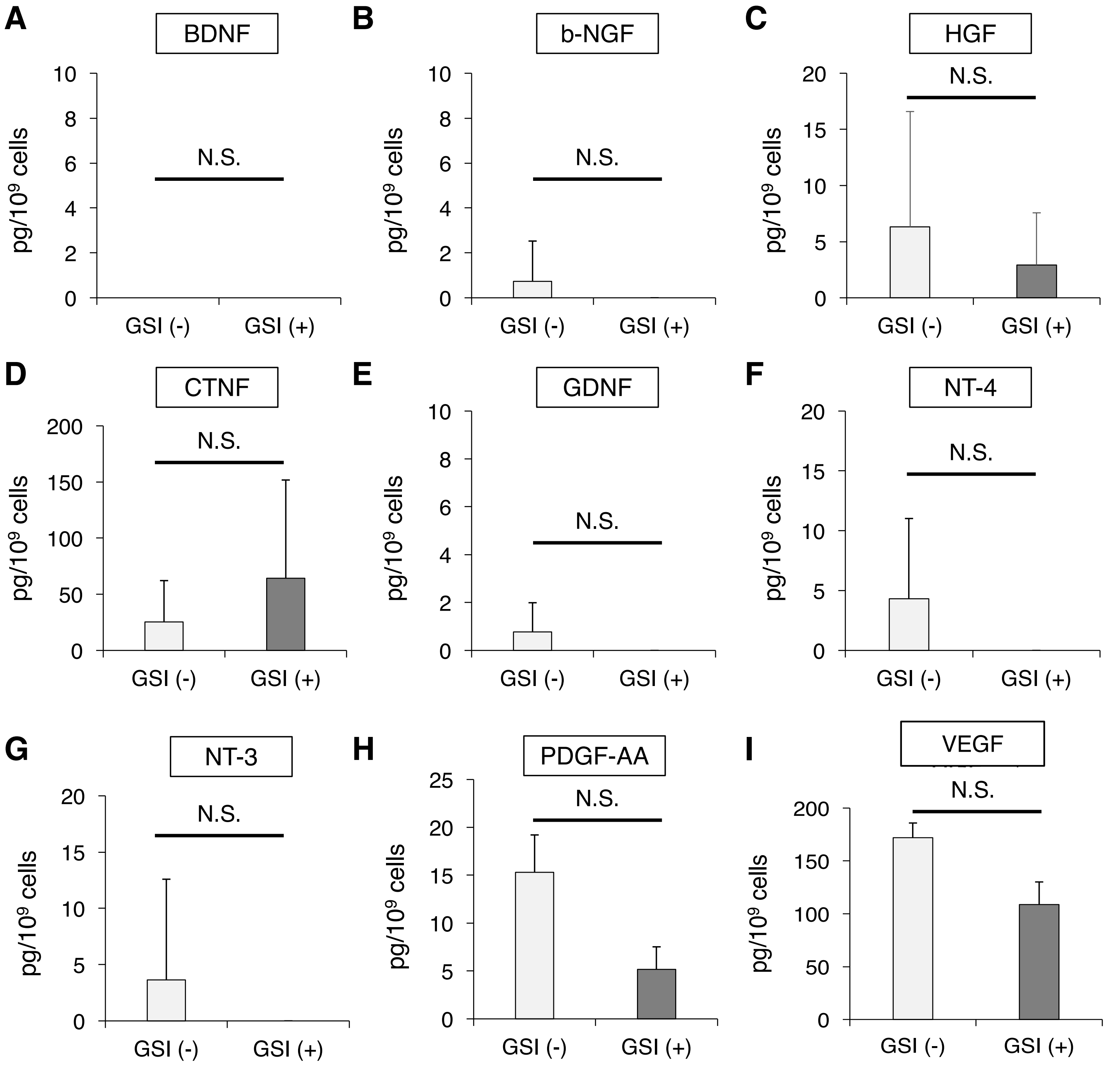
A
BDNF
B
b-NGF
C
HGF
D
CTNF
E
GDNF
F
NT-4
G
NT-3
H
PDGF-AA
I
VEGF
N.S.
pg/10⁹ cells
GSI (-)
GSI (+)

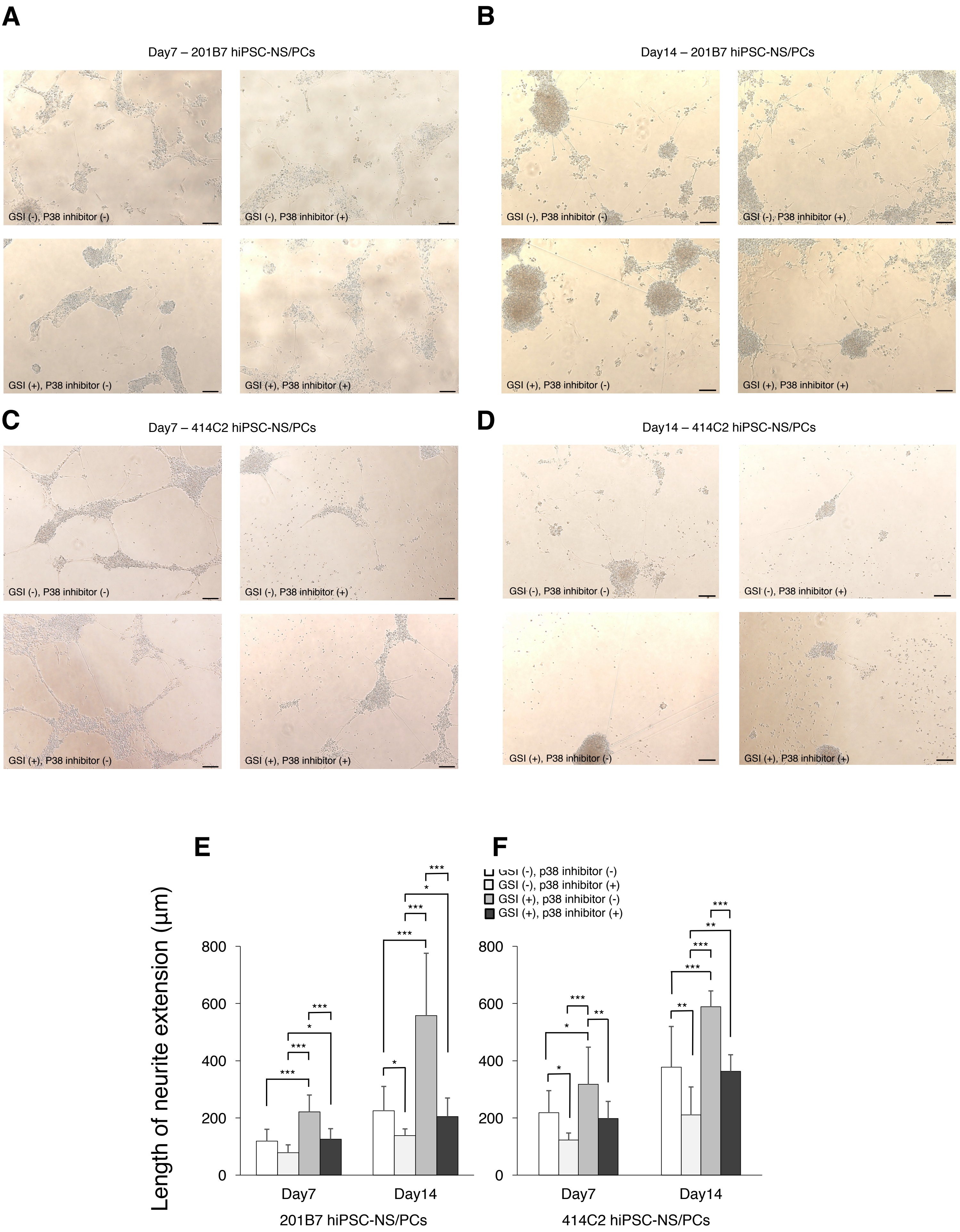

A
Day7 – 201B7 hiPSC-NS/PCs
GSI (-), P38 inhibitor (-)
GSI (-), P38 inhibitor (+)
GSI (+), P38 inhibitor (-)
GSI (+), P38 inhibitor (+)
B
Day14 – 201B7 hiPSC-NS/PCs
GSI (-), P38 inhibitor (-)
GSI (-), P38 inhibitor (+)
GSI (+), P38 inhibitor (-)
GSI (+), P38 inhibitor (+)
C
Day7 – 414C2 hiPSC-NS/PCs
GSI (-), P38 inhibitor (-)
GSI (-), P38 inhibitor (+)
GSI (+), P38 inhibitor (-)
GSI (+), P38 inhibitor (+)
D
Day14 – 414C2 hiPSC-NS/PCs
GSI (-), P38 inhibitor (-)
GSI (-), P38 inhibitor (+)
GSI (+), P38 inhibitor (-)
GSI (+), P38 inhibitor (+)
E
F
(-), p38 inhibitor (-)
GSI (-), p38 inhibitor (+)
GSI (+), p38 inhibitor (-)
GSI (+), p38 inhibitor (+)
Length of neurite extension (μm)
800
600
400
200
0
Day7
Day14
201B7 hiPSC-NS/PCs
414C2 hiPSC-NS/PCs

Supplemental Figure Legends

Figure S1. Detection of Bioluminescence and Fluorescence Signals in Lentivirally Transfected hiPSC-NS/PCs *in vitro*

(A) Bioluminescence imaging was used to detect bioluminescence signals in various numbers of hiPSC-NS/PCs (0, 1×10^2, 1×10^3, 1×10^4, 1×10^5, and 5×10^5 cells per well).

(B) BLI significantly find out a direct linear correlation between cell numbers and photon counts *in vitro* (n = 5 independent experiments).

The data are presented as the means ± SEM. BLI; bioluminescence imaging

Figure S2. Grafted cells were extended from rostral to caudal site over the epicenter of chronically injured spinal cord

(A) In the GSI (+) group, representative immunohistological images of STEM121 positive cells in the chronically injured spinal cord at 84 days after transplantation. Scale bars, 1,000 mm.

(B) and (C) Boxed area in (A).

Figure S3. Neurotrophic Factors Are Not Secreted into the Culture Medium of GSI-treated hiPSC-NS/PCs

(A) – (I) Quantification of secreted cytokines from GSI-treated hiPSC-NS/PCs and conventional hiPSC-NS/PCs. GSI-treated hiPSC-NS/PCs showed no significant difference in the secretion of various neurotrophic factors into the culture medium compared with conventional hiPSC-NS/PCs according to the Wilcoxon rank-sum test (n = 3 independent experiments). N.S. = Non-significant. The data are presented as the mean ± SEM.

Figure S4. Quantification of Neurite Extension from hiPSC-NS/PCs Treated with GSI Alone, a p38 MAPK Inhibitor Alone or a Combination of Both

(A) - (D) Micrographs showing representative neurite outgrowth from hiPSC-NS/PCs treated with GSI alone, a p38 MAPK inhibitor alone or both at 7 and 14 days after neuronal differentiation. Scale bars, 100 μm.

(E) and (F) Length of neurite extension from each cell measured in 10 separate fields using a microscope and compared among each group (n = 5 independent experiments).

$^{*}p < 0.05$, $^{**}p < 0.01$ and $^{***}p < 0.001$ according to a one-way ANOVA with the Tukey-Kramer test.

Supplemental Experimental Procedures

Treatment of hiPSC-NS/PCs with GSI

The small molecule GSI, N-[N-(3,5-difluorophenacetyl)-l-alanyl]-S-phenylglycine t-butyl ester (DAPT; Sigma-Aldrich, St. Louis, MO, USA, D5942), is a potent nontransition-state analog inhibitor of g-secretase that is thought to interact with the same active site between presenilin-1 heterodimers in gamma-secretase complexes. DAPT was dissolved in DMSO at a final concentration of 10 mM. The highest concentration of this molecule that effectively inhibited the division of hiPSC-NS/PCs without precipitating in culture medium or producing toxic effects was determined in preliminary experiments. For cell transplantation assays, the hiPSC-NS/PCs were used after the fifth passage.

Histological analyses

Animals were anesthetized and transcardially euthanized with 0.1 M PBS containing 4% paraformaldehyde

(PFA) at 84 days after transplantation. Their spinal cords were then removed, postfixed overnight in 4% PFA, soaked overnight in 10% sucrose, followed by 30% sucrose, embedded in Optimal Cutting Temperature (O.C.T) compound (Sakura Finetechnical Co., Ltd., Tokyo, Japan), frozen, and sectioned along the sagittal and axial planes at a 14 μm thickness on a cryostat (CM3050S, Leica Microsystems, Wetzlar, Germany). Spinal cord sections were histologically evaluated by staining with HE and LFB and through immunohistochemistry (IHC). Tissue sections were stained with the following primary antibodies for IHC: anti-pan-ELAVL (Hu) (human IgG, 1:1000, a gift from Dr. Robert Darnell; The Rockefeller University, New York, NY, USA), anti-GFAP (rabbit IgG, 1:200; Dako, Carpinteria, CA, USA, Z0334), anti-APC CC-1 (mouse IgG, 1:300; Abcam, Cambridge, UK, ab16794), anti-human-specific Nestin protein (rabbit IgG, 1:300; described previously (Kanemura et al., 2002)), anti-Ki67 (rabbit IgG, 1:200; Leica Biosystems, Wetzlar, Germany), anti-human nuclear antigen (anti-HNA, mouse IgG, 1:200; Chemicon, Temecula, CA, USA, MAB1281), anti-neurofilament 200 kDa (NF-H, mouse IgG, 1:200; Merck Millipore, Billerica, MA, USA, MAB5266), anti-5-hydroxytrypamine (5HT; goat IgG, 1:200; Immunostar, Hudson, WI, USA, 20080), anti-p38 MAPK (p38; rabbit IgG, 1:200; Cell Signaling Technology, Danvers, MA, USA, 8690L), anti-phospho-p38 MAPK (Pp38; rabbit IgG, 1:200; Cell Signaling Technology, Danvers, MA, USA, 4511L), anti-GAD67 (mouse IgG2a, 1:200; Merck Millipore, Billerica, MA,

USA, MAB5406), anti-VGLuT1 (rabbit IgG, 1:100; Abcam, Cambridge, UK, ab72311), anti-human cytoplasm (STEM121; mouse IgG1, 1:300; Takara Bio, Kusatsu, Japan, Y40410), anti-β-tubulin (Tuj1; mouse IgG, 1:300; Sigma-Aldrich, St. Louis, MO, USA, T8660), anti-Bsn (mouse IgG, 1:200; Stressgen, ADI-VAM-PS003), anti-hSyn (Mouse IgG, 1:200; Chemicon, Temecula, CA, USA, MAB332), anti-PSD95 (rabbit, 1:100; Cell Signaling Technology, Danvers, MA, USA, 2507), and anti-Gephyrin (rabbit IgG, 1:250; Synaptic Systems, Göttingen, Germany, 147008). Nuclei were stained with Hoechst 33258 (10 µg/ml, Sigma-Aldrich, St. Louis, MO, USA). For IHC of RtST fibers, a streptavidin secondary antibody (Alexa Fluor 555 conjugate, 1:500; Thermo Fisher, Waltham, MA, USA, S32355) was used. Samples were visualized using a fluorescence microscope (BZ-X710; Keyence Co., Osaka, Japan) or a confocal laser-scanning microscope (LSM 700, Carl Zeiss, Jena, Germany). The numbers of marker-positive cells, such as HNA-, Ki67-, Nestin-, pan-ELAVL (Hu)-, GFAP-, and APC-positive cells, as well as NF-H-, 5-HT-, and BDA-labeled RtST-positive fibers were counted in each section (n = 5 per group).
